# A Variational Bayes Approach to the Analysis of Occupancy Models

**DOI:** 10.1371/journal.pone.0148966

**Published:** 2016-02-29

**Authors:** Allan E. Clark, Res Altwegg, John T. Ormerod

**Affiliations:** 1 Department of Statistical Sciences, University of Cape Town, Private Bag X3, Rondebosch 7701, Cape Town, South Africa; 2 Centre for Statistics in Ecology, Environment and Conservation (SEEC), University of Cape Town, Cape Town, South Africa; 3 School of Mathematics and Statistics, University of Sydney, Sydney, Australia; 4 ARC Centre of Excellence for Mathematical & Statistical Frontiers, Melbourne, Australia; Field Museum of Natural History, UNITED STATES

## Abstract

Detection-nondetection data are often used to investigate species range dynamics using Bayesian occupancy models which rely on the use of Markov chain Monte Carlo (MCMC) methods to sample from the posterior distribution of the parameters of the model. In this article we develop two Variational Bayes (VB) approximations to the posterior distribution of the parameters of a single-season site occupancy model which uses logistic link functions to model the probability of species occurrence at sites and of species detection probabilities. This task is accomplished through the development of iterative algorithms that do not use MCMC methods. Simulations and small practical examples demonstrate the effectiveness of the proposed technique. We specifically show that (under certain circumstances) the variational distributions can provide accurate approximations to the true posterior distributions of the parameters of the model when the number of visits per site (*K*) are as low as three and that the accuracy of the approximations improves as *K* increases. We also show that the methodology can be used to obtain the posterior distribution of the predictive distribution of the proportion of sites occupied (PAO).

## Introduction

Bayesian analysis is a coherent statistical paradigm whereby prior information regarding the research area is blended with that of information obtained from the observed data [1]. Subjective prior information is *elicited* either from expertise in the field or based on prior research (meta analyses). Informative priors are increasingly being used in ecology ([2, 3]) and even in the absence of prior information many ecologists are using Bayesian methods [4].

One class of model that is often analysed in a Bayesian way is the occupancy model [5]. The single season occupancy model was formulated by using ideas borrowed from closed population mark-recapture models. In this model *n* sites are visited a number of times (*K*) in order to estimate the occupancy (***ψ***) and detection probability (denoted throughout as ***d***) of a species associated with each site. (The term *detection probability* should be read as *conditional detection probability* throughout the text.) These methods are particularly useful when studying the range dynamics of various animal species and have extensively been applied in the ecological literature (see [6, 7] and [8] for some examples). The model has been formulated as a hierarchical Bayesian model which has lead to numerous extensions of the single season occupancy model ([9, 10] and [11]).

Many papers have investigated the statistical properties of the estimators of the single season occupancy model. The first of these developed a maximum likelihood formulation of the model and investigated the properties of the estimators for the occupancy and detection probabilities using simulations. They assume that the parameters of the model are constant for all sites although also consider incorporating covariates in the model. They found that when *d* ≥ 0.3, the parameter estimates of the occupancy probability were reasonably unbiased when *K* ≥ 5 while when *K* = 2, a detection probability of at least 0.5 is required to provide a reasonable estimate of *ψ*. They also found that when the true detection probability is low that $\hat{\psi }$ tends to 1 [5]. Numerous authors have found similar results regarding boundary problems ([12, 13] and [14]) although it has been argued that boundary parameter estimates are rare but could be observed in small data sets [13].

Moreno and Lele investigated the small sample properties of the maximum likelihood estimators [15]. They note that ‘When detection or occupancy probability is small or when the number of sites and number of visits per site is small, maximum likelihood estimators (MLE) of site occupancy parameters have large biases, are numerically unstable, and the corresponding confidence intervals have smaller than nominal coverage.’ They proposed a penalized maximum likelihood method which performed adequately for small sample sizes. Recently, their study has been extended by considering three different penalized likelihood type models [14]. They found that the penalized methods performed well and suggested that ‘fully Bayesian methods would be competitive’.

Here, we develop Variational Bayes (VB) approximations to the posterior distribution of the parameters of a single-season site occupancy model. One big advantage of the methods developed here is the fact that they could be applied to cases where the researcher has informative priors and might not want to rely on the use of the MLE method. In that situation, Markov Chain Monte Carlo (MCMC) methods were so far the only methods available for fitting occupancy models in a Bayesian analysis. However, for big data sets, MCMC methods can be too slow to be useful. Admittedly the potential computational efficiencies accrued from using a VB algorithm compared to the MLE method would possibly only apply when fitting more complicated occupancy type models. We view our contribution as a first step towards developing similar methods for more complicated occupancy models (e.g., the inclusion of site-specific random effects, spatial occupancy models and dynamic occupancy models).

The proportion of occupied sample locations ($\bar{z}=1/n\sum_{i}z_{i}$ where *z*_*i*_ is the occupancy state for site *i*) is a derived parameter of interest in many studies ([9, 16]). Although frequentist methods can be used to estimate $\bar{z}$, the calculation of a valid confidence interval for $\bar{z}$ is problematic for the frequentist. The same holds true for prediction of occupancy status in species distribution models [17]. We show (via simulations as well as using practical examples) that the VB approximations can be used to accurately obtain prediction intervals for latent state variables (e.g., occupancy states ***z***) or for functions of these state variables by simulating from the VB posterior distributions.

This paper commences with a brief discussion of Variational Bayes (VB). Thereafter, a VB implementation of a particular occupancy model is developed in Section 1.2. In Section 2.1 the results of a short simulation study are presented while in Section 2.2 we analyse site occupancy data of five bird species to illustrate the usefulness of the VB technique developed. A list of some of the notations and distribution theory used in the text can be found in S1 Text.

## 1 Material and Methods

### 1.1 A brief introduction to Variational Bayes (VB)

Variational Bayes is used to approximate posterior distributions obtained when undertaking Bayesian analysis and could be useful in many ecological applications.

In what follows let ***θ*** be a vector of parameters of a statistical model, *π*(***θ***) be a prior distribution for these parameters and ***y*** be a random variable. In the context of this article, ***θ*** are the parameters of a single-season occupancy model while ***y*** represents detection-nondetection data used to fit an occupancy model. Further, suppose that a posterior distribution *π*(***θ***|***y***) is not analytically tractable and that analytical expressions for its posterior moments do not exist. In probability theory the Kullback-Leibler (KL) divergence provides a measure of a difference between two probability distributions [18]. When the two distributions being compared are exactly the same the divergence measure is equal to zero while when they are different the divergence measure is positive.

The VB method approximates a posterior distribution by using a distribution *q*(***θ***) which is obtained by minimizing the Kullback-Leibler (KL) divergence between *q*(***θ***) and *π*(***θ***|***y***) [18]. The KL divergence is

$$\begin{array}{c} KL(q\left(\theta \right)||p(\theta |y))=\int q\left(\theta \right)\text{ln}\;\left(\frac{q\left(\theta \right)}{p(\theta |y)}\right)\;d\theta =\int q\left(\theta \right)\text{ln}\;\left(\frac{q\left(\theta \right)}{p\left(\theta ,y\right)}\right)\;d\theta +\text{ln}p\left(y\right) \end{array}$$ (1)

where *p*(***y***) is the marginal likelihood, *p*(***y***, ***θ***) is the joint likelihood of the data and the parameter vector ***θ*** with

$$\begin{array}{c} L\left(q\left(\theta \right)\right)=\int q\left(\theta \right)\ln\left(\frac{p(y,\theta )}{q(\theta )}\right)\;d\theta . \end{array}$$ (2)

Since *KL* (*q*(***θ***)||*p*(***θ***|***y***)) ≥ 0, ln *p*(***y***) ≥ *L*(*q*(***θ***)) for every *q*(***θ***) and minimising *KL* (*q*(***θ***)||*p*(***θ***|***y***)) is equivalent to maximising *L*(*q*(***θ***)). Often it is assumed that *q*(***θ***) can be factorized as a product of simple probability distributions as *q*(***θ***) = ∏_*i*_ *q*(*θ*_*i*_) where each of the *q*(*θ*_*i*_) are iteratively estimated as $\ln q\left(\theta_{i}\right)\propto E_{-\theta_{i}}(\ln p(y,\theta ))$. Here $E_{-\theta_{i}}$ denotes an expectation with respect to the density ∏_*j* ≠ *i*_ *q*(*θ*_*j*_). An alternate method of obtaining *q*(***θ***) involves making an assumption regarding its parametric form. The parameters of this distribution are obtained my maximising *L*(*q*(***θ***)) [19].

VB is often used as an alternative to Markov chain Monte Carlo (MCMC) methods since the method can be much faster to implement since in most applications *q*(*θ*_*i*_) will be of a known simple form ([20–22]). Variational approximations to posterior distributions can accurately estimate the posterior mean of the parameters, although the posterior variances of some of the parameters might be underestimated ([23, 24]). Although this problem is context specific the estimate of the posterior variance is asymptotically valid for linear models [25]. As a solution the variational covariance matrix is often replaced by the inverse of the Fishers’ information matrix [23]. Alternately the non-parametric bootstrap could be used to provide interval estimates of the parameters [26].

### 1.2 VB applied to single season occupancy models

In a single season occupancy model *n* sites are visited ***K*** times in order to estimate the occupancy (***ψ***) and detection (***d***) probability of a species associated with each site. Each site could be surveyed a different amount of times such that ***K*** = (*K*_1_, *K*_2_, … , *K*_*n*_)^*T*^ where *K*_*i*_ represents the number of surveys undertaken to site *i* and ***d*** is a ragged matrix with dimensions determined by ***K***. The total number of site visits undertaken is defined as *N* = ∑_*i*_ *K*_*i*_.

The data collected at each site are represented as an *N* dimensional vector $y={\left(y_{1}^{T},\ldots ,y_{n}^{T}\right)}^{T}$, where each of the ***y***_*i*_ denotes the vector of detections and nondetections for site *i*. A 0 in the vector ***y***_*i*_ indicates that the species was not observed at the *i*^*th*^ site during a particular visit while a 1 indicates that the species was observed at the particular site during a particular visit. Let the vector ***z*** represent the true species occupancy at the sites considered. Since we are using a single season model, ***z*** is assumed to be constant across the season. ***z*** is partially observed, i.e. *z*_*i*_ = 1 if the species occupies site *i* and *z*_*i*_ = 0 if it does not occupy site *i*. We know *z*_*i*_ = 1 if the species is observed at site *i* during any of the visits since we assume that there are no false identifications of individuals. If the species is however not observed at site *i*, *z*_*i*_ could equal 0 or 1 since we are uncertain about whether the species actually occurs at that site. We treat ***y***_*i*_ as a row vector and is of dimension 1 × *K*_*i*_ while ***z*** is of dimension *n* × 1.

The single season occupancy models can be represented using the following hierarchical model [9]

$$\begin{array}{c} \;z_{i}|\psi_{i}\;\sim \;Bernoulli(\psi_{i})\\ y_{i,j}|z_{i},d_{i,j}\;\sim \;Bernoulli(z_{i}d_{i,j}) \end{array}$$

for all sites *i* = 1, … , *n*; for all visits *j* = 1, … , *K*_*i*_. *ψ*_*i*_ = *Pr*(*z*_*i*_ = 1) denotes the probability that the species occurs at site *i* while *p*_*i*, *j*_ = *Pr*(*y*_*i*, *j*_ = 1|*z*_*i*_ = 1) denotes the conditional probability of detecting the species during the *j*^*th*^ visit of site *i* given that the species is present at site *i*. The occupancy probabilities and the detection probabilities can be estimated using either maximum likelihood [5], penalized maximum likelihood [15] or Bayesian methods [9]. In what follows we develop a VB approach to estimating these quantities.

Additional covariate data collected at each of the sites are used to estimate the site occupancy and detection probabilities. Specifically we assume that we have *r* occupancy and *s* detection covariates. We further assume that we have no missing values in these covariates. Formally we let ***W*** and ***X*** be the design matrices for the detection and occupancy effects respectively, with dimensions *N* × *s* and *n* × *r*. Correspondingly, let ***α*** and ***β*** be the detection and occupancy effects with dimensions *s* × 1 and *r* × 1 respectively. The matrix ***W*** is constructed by row-binding the detection covariates at the different locations and for different visits one below each other such that $W={\left({\underline{w}}_{1}^{T},\ldots ,{\underline{w}}_{n}^{T}\right)}^{T}$ where each of the ${\underline{w}}_{i}$ matrices are of dimension *K*_*i*_ × *s* with ${\underline{w}}_{i}={\left(w_{i,1}^{T},w_{i,2}^{T},\ldots ,w_{i,K_{i}}^{T}\right)}^{T}$.

The occupancy and detection probabilities at the various sites for all visits are modelled using the following logistic link functions

$$\begin{array}{cc} \psi_{i} & ={\left(1+\text{exp}\left(-x_{i}\beta \right)\right)}^{-1}\\ d_{i,j} & =\;{\left(1+\text{exp}\left(-w_{i,j}\alpha \right)\right)}^{-1}, \end{array}$$

It can be shown that the conditional likelihood of the data and the true occupancy variables is

$$\begin{array}{c} p\left(y,z|\alpha ,\beta \right)=\prod_{i=1}^{n}\psi_{i}^{z_{i}}{\left(1-\psi_{i}\right)}^{1-z_{i}}\prod_{j=1}^{K_{i}}{\left(z_{i}d_{i,j}\right)}^{y_{i,j}}{\left(1-z_{i}d_{i,j}\right)}^{1-y_{i,j}}. \end{array}$$

We now assume that the prior distribution for ***α*** and ***β*** are multivariate Gaussian distributions (denoted as *π*(***α***, ***β***)) with parameters $\mu_{\alpha }^{0}$, $\Sigma_{\alpha }^{0}$ and $\mu_{\beta }^{0}$, $\Sigma_{\beta }^{0}$ respectively. We further assume that the **variational approximate distribution** of *π*(***α***, ***β***, ***z***) is of the form *q*(***α***, ***β***, ***z***) = *q*(***α***, ***β***)∏_*i*_ *q*(*z*_*i*_) where each of the *q*(*z*_*i*_) are Bernoulli distributed with success probability (*sp*)_*i*_. Under this restriction *q*(***α***, ***β***) can be factorized into two separate factors *q*(***α***) and *q*(***β***) with

$$q(\alpha )\;\propto \;\text{exp}\left(y^{T}\tilde{P}W\alpha -{\tilde{p}}^{T}b\left(W\alpha \right)+\text{ln}\pi (\alpha )\right)$$ (3)

$$q(\beta )\;\propto \;\text{exp}\left(p^{T}X\beta -1_{n}^{T}b\left(X\beta \right)+\text{ln}\pi (\beta )\right),$$ (4)

where $\tilde{p}=E_{q(\alpha ,\beta )}\left(\tilde{z}|y\right)$, $\tilde{P}=\text{diag}\left(\tilde{p}\right)$, $p=E_{q(\alpha ,\beta )}\left(z|y\right)$ and *b*(*x*) = ln(1 + exp(*x*)). Here $\tilde{z}={\left(z_{1}1_{K_{1}}^{T},\ldots ,z_{n}1_{K_{n}}^{T}\right)}^{T}$. Refer to S1 Appendix for a derivation of the above results.

The normalization constant of *q*(***α***, ***β***) is not known analytically and thus *q*(***α***) and *q*(***β***) are not of a known type. We attempt to approximate the posterior distribution of (***α***, ***β***) using two different methods. In the first method we approximate the variational distribution by using a Laplace approximation to Eqs (3) and (4) and thus assume that the variational distributions are multivariate Gaussian with parameters ***μ*_*α*_**, **Σ_*α*_** and ***μ*_*β*_**, **Σ_*β*_** respectively; while in the second method we employ a tangent based approximation to *b*(***Wα***) and *b*(***Xβ***) to obtain approximations to *q*(***α***) and *q*(***β***) respectively.

Once we have obtained approximations to *q*(***α***, ***β***) it then follows that the *q*-densities, *q*(*z*_*i*_|***y***_*i*_ = **0**)∀*i*, is Bernoulli distributed with success probability (1 + exp(−*c*_*i*_))^−1^. The approximate conditional occupancy probabilities for all sites can then be calculated for the two methods (denoted as ‘*L*’ and ‘*T*’ respectively) using

$$c_{i}^{\left(L\right)}\;=\;x_{i}\mu_{\beta }-1_{K_{i}}^{T}E_{q(\alpha )}\left(b\left({\underline{w}}_{i}\alpha \right)\right)$$ (5)

$$d_{i}\;=\;{\underline{w}}_{i}^{T}\text{diag}(A({\underline{a}}_{i})){\underline{w}}_{i}\left(\Sigma_{\alpha }+\mu_{\alpha }\mu_{\alpha }^{T}\right)$$

$$c_{i}^{\left(T\right)}=x_{i}\mu_{\beta }+1_{K_{i}}^{T}C({\underline{a}}_{i})-\frac{1}{2}1_{K_{i}}^{T}{\underline{w}}_{i}\mu_{\alpha }+\text{tr}\left(d_{i}\right).$$ (6)

Here $b({\underline{w}}_{i}\alpha )$ is a vector of length *K*_*i*_ such that

$$\begin{array}{c} \langle b\left({\underline{w}}_{i}\alpha \right)\rangle ={\left(\langle b\left(w_{i,1}\alpha \right)\rangle ,\langle b\left(w_{i,2}\alpha \right)\rangle ,\ldots ,\langle b\left(w_{i,K_{i}}\alpha \right)\rangle \right)}^{T} \end{array}$$

The parameters of the variational distributions are all dependent on one another and can be computed using an iterative scheme such as that given in Algorithm 1 and Algorithm 2. A detailed description of aspects of the above derivations can be found in the supplemental information to this paper. In particular, the quantities used to calculate $c_{i}^{\left(T\right)}$ can be found in S2 Appendix while an explanation regarding the stopping rule for both algorithms is described in S3 Appendix.

### 1.3 SIMULATION STUDY

In the following simulation study we investigate some of the properties of the VB method and investigate whether it could be used to produce *statistically valid inference*. We specifically focus on the frequentist properties of the posterior mean parameters of the VB distribution of ***α*** and ***β***. This task is undertaken by *empirically* comparing the coverage probability and credibility/confidence intervals of ***α*** and ***β*** associated with the two VB methods developed and comparing these to the same statistics obtained using MCMC and maximum likelihood. We calculate credibility intervals for the Bayesian methods and confidence intervals for the MLE method and focus particularly on the 95% credibility or confidence intervals.

**Algorithm 1 Iterative scheme for obtaining the parameters of the optimal density of *q*(*α*, *β*) using the Laplace approximation.**

1. Initialize ***μ*_*α*_**, **Σ_*α*_**, ***μ*_*β*_**, **Σ_*β*_**

2. Cycle:

 2.1 Cycle:

   $g_{1}\leftarrow W^{T}(\tilde{P}y-\tilde{p}⊙b^{\prime }(W\mu_{\alpha }))-{\left(\Sigma_{\alpha }^{0}\right)}^{-1}(\mu_{\alpha }-\mu_{\alpha }^{0})$

   $g_{2}\leftarrow X^{T}(p-b^{\prime }(X\mu_{\beta }))-{\left(\Sigma_{\beta }^{0}\right)}^{-1}(\mu_{\beta }-\mu_{\beta }^{0})$

   $\Sigma_{11}\leftarrow {\left(W^{T}\text{diag}\left(\tilde{p}⊙b^{\prime \prime }\left(\;W\;\mu_{\alpha }\right)\right)W+{\left(\Sigma_{\alpha }^{0}\right)}^{-1}\right)}^{-1}$

   $\Sigma_{22}\leftarrow {\left(X^{T}\text{diag}\left(b^{\prime \prime }\left(\;X\;\mu_{\beta }\right)\right)X+{\left(\Sigma_{\beta }^{0}\right)}^{-1}\right)}^{-1}$

   ***μ*_*α*_** ← ***μ*_*α*_** + **Σ**_11_ ***g***_1_ and ***μ*_*β*_** ← ***μ*_*β*_** + **Σ**_22_ ***g***_2_

  until the Newton-Raphson algorithm converges.

 2.2 Calculate conditional occupancy probabilities for all sites where ***y***_*i*_ = **0** using Eq (5). Note that (*sp*)_*i*_ = 1 for all sites where ${\underline{y}}_{i}\neq \underline{0}$.

until the change in $E_{q}\left(\ln\underline{p}\right)-E_{q}\left(\ln q\left(\alpha ,\beta \right)\right)-E_{q}\left(\ln q\left(z\right)\right)$ becomes negligible. (≤ 10^−6^)

**Algorithm 2 Iterative scheme for obtaining the parameters of the optimal density of *q*(*α*, *β*) using the tangent based method.**

1. Initialize ***μ***_***α***_, **Σ_*α*_**, ***μ*_*β*_**, **Σ_*β*_**, ***a***_*N*_ > 0 and *b*_*N*_ > 0.

2. Cycle:

 2.1 Calculate the conditional occupancy probabilities for all sites where ***y***_*i*_ = **0** using Eq (6). Note that (*sp*)_*i*_ = 1 for all sites where ${\underline{y}}_{i}\neq \underline{0}$.

 2.2 Set $\mu_{\alpha }\leftarrow B_{1}^{-1}B_{2}^{T}$, $\mu_{\beta }\leftarrow D_{1}^{-1}D_{2}^{T}$, $\Sigma_{\alpha }\leftarrow B_{1}^{-1}$ and $\Sigma_{\beta }\leftarrow D_{1}^{-1}$ with

   $B_{1}\leftarrow {\left(\Sigma_{\alpha }^{0}\right)}^{-1}-2W^{T}\text{diag}\left(A\left(a\right)⊙\tilde{p}\right)W$

   $B_{2}\leftarrow {\left(\tilde{P}y-\frac{1}{2}\tilde{p}\right)}^{T}\;W+{\left(\mu_{\alpha }^{0}\right)}^{T}{\left(\Sigma_{\alpha }^{0}\right)}^{-1}$

   $D_{1}\leftarrow {\left(\Sigma_{\beta }^{0}\right)}^{-1}-2X^{T}\text{diag}\left(A\left(b\right)\right)X$

   $D_{2}\leftarrow {\left(p-\frac{1}{2}1_{n}\right)}^{T}\;X+{\left(\mu_{\beta }^{0}\right)}^{T}{\left(\Sigma_{\beta }^{0}\right)}^{-1}$

 2.3 Calculate the ‘variational parameters’. Refer to S2 Appendix.

until the change in $E_{q}\left(\ln\underline{p}\right)-E_{q}\left(\ln q\left(\alpha ,\beta \right)\right)-E_{q}\left(\ln q\left(z\right)\right)$ becomes negligible. (≤ 10^−6^)

The accuracy of the VB approximations to the posterior distribution obtained through MCMC is also assessed. This is undertaken by calculating $\text{acc}\left(x\right)=1-\frac{1}{2}\int \left|q\left(x\right)-q_{MCMC}\left(x\right)\right|dx$. The *acc*(*x*) measure lies between 0 and 1 with a value of 1 indicating a perfect approximation and a value close to 0 indicating a poor approximation by the variational distribution to the true posterior distribution.

Occupancy models are often used to assess the predictive distribution of the proportion of occupied sites defined as $\text{PAO}=\frac{1}{n}\sum_{i=1}^{n}z_{i}$. We thus investigate the posterior approximation of the PAO using the Laplace VB posterior approximation method. These can easily be obtained by sampling from the VB posterior distribution for each *z*_*i*_ in turn to construct the PAO statistic. To assess the VB approximations the acc(*x*) statistic was used.

We consider 32 simulation settings. The number of sites (*n*) are set to 50 and 100 while the number of visits to each site (*K*) are set to 2, 3, 4 and 5 respectively. The following combinations of the regression coefficients were used: 1. ***α*** = [0, 1.75]^*T*^, ***β*** = [−1.85, 2.5]^*T*^; 2. ***α*** = [1.35, 1.75]^*T*^, ***β*** = [−1.85, 2.5]^*T*^; 3. ***α*** = [0, 1.75]^*T*^ ***β*** = [−0.1, 2.5]^*T*^ and 4. ***α*** = [1.35, 1.75]^*T*^ ***β*** = [−0.1, 2.5]^*T*^. These parameter values ensure an approximate average detection and occupancy probability among the sites of (0.5, 0.3), (0.7, 0.3), (0.5, 0.5) and (0.5, 0.7) respectively. We have not considered any cases where the detection and occupancy probabilities are lower than 0.3 since in these cases data sets are expected to be very sparse which requires many site visits in order to undertake useful statistical inference [5].

The occupancy regression covariate was obtained by standardizing a Uniform(−2, 2) random variable while the detection covariate was obtained by standardizing a Uniform(−5, 5) random variable. Each of these variables were transformed to have a zero mean and a standard deviation of one. The following parameter vectors were used to specify the prior distribution of the parameters: $\mu_{ı}^{0}={\left[0,0\right]}^{T}$, $\Sigma_{ı}^{0}=\text{diag}\left[1000,1000\right]$ for ***i*** = ***α***, ***β***.

Each simulation setting was replicated 350 times. All calculations were undertaken using R 3.3.1 [27]. Numerical optimizations were performed using the the BFGS method of the R function *optim*; MCMC sampling was undertaken using the R package R2jags [28] in combination with JAGS 3.4.0 [29] while all variational approximations were performed using the authors’ code. 100000 posterior samples were obtained for each MCMC simulation. The first 25000 samples were discarded as burn-in samples while the remaining 75000 samples were retained. Prior experimentation using the MCMC algorithm indicated that 25000 iterations are enough to ensure that the Markov chains would converge to the stationary distributions. The posterior samples were not thinned [30].

## 2 Results

### 2.1 SIMULATION RESULTS

Table 1 contains a summary of some of the results of the simulation study. For each value of *K* we tabulate the median coverage probability and credibility/confidence interval width of ***α*** and ***β*** associated with the four estimation procedures considered here. The medians are calculated across the different occupancy and detection probability combinations for fixed values of *n* and *K*.

**Table 1:** The median coverage probability and credibility/confidence interval widths of the covariate effects for the single season occupancy model.

			K							
			2		3		4		5	
n	Parameters	Method	Coverage	Width	Coverage	Width	Coverage	Width	Coverage	Width
50	*α*_0_	Laplace	0.929	2.006	**0.944**	1.619	**0.956**	1.393	**0.962**	1.221
		Tangent	0.793	**1.51**	0.838	**1.22**	0.841	**1.05**	0.854	**0.93**
		MLE	**0.96**	2.675	0.964	1.869	0.962	1.509	0.968	1.28
		MCMC	0.95	3.219	0.95	2.203	0.95	1.769	0.95	1.284
	*α*_1_	Laplace	**0.944**	2.452	**0.959**	1.986	**0.96**	1.704	0.966	1.493
		Tangent	0.76	**1.642**	0.787	**1.31**	0.812	**1.127**	0.803	**0.996**
		MLE	0.962	2.926	0.97	2.215	0.968	1.81	**0.963**	1.55
		MCMC	0.95	3.295	0.95	2.407	0.95	2.02	0.95	1.635
	*β*_0_	Laplace	**0.936**	1.994	**0.952**	2.019	**0.963**	2.032	**0.964**	2.022
		Tangent	0.75	**1.345**	0.821	**1.348**	0.845	**1.349**	0.841	**1.349**
		MLE	0.973	2.776	0.974	2.505	0.974	2.416	0.97	2.322
		MCMC	0.95	4.716	0.95	3.452	0.95	2.95	0.95	2.553
	*β*_1_	Laplace	0.92	2.656	**0.952**	2.679	**0.952**	2.705	**0.952**	2.71
		Tangent	0.666	**1.493**	0.744	**1.494**	0.733	**1.498**	0.723	**1.498**
		MLE	**0.954**	4.11	0.963	3.578	0.964	3.419	0.969	3.28
		MCMC	0.95	6.684	0.95	5.41	0.95	4.319	0.95	3.822
100	*α*_0_	Laplace	0.907	1.402	0.932	1.103	**0.946**	0.944	0.942	0.852
		Tangent	0.764	**1.048**	0.823	**0.842**	0.858	**0.726**	0.826	**0.651**
		MLE	**0.96**	1.784	**0.96**	1.24	**0.954**	1.008	**0.95**	0.88
		MCMC	0.95	2.01	0.95	1.445	0.95	0.986	0.95	0.924
	*α*_1_	Laplace	**0.949**	1.728	0.942	1.351	**0.949**	1.143	**0.953**	1.033
		Tangent	0.76	**1.126**	0.768	**0.905**	0.806	**0.778**	0.79	**0.696**
		MLE	0.968	2.025	**0.949**	1.444	0.947	1.182	**0.953**	1.05
		MCMC	0.95	2.235	0.95	1.57	0.95	1.222	0.95	1.11
	*β*_0_	Laplace	0.928	1.459	**0.957**	1.478	**0.95**	1.475	**0.95**	1.484
		Tangent	0.723	**0.954**	0.766	**0.957**	0.792	**0.956**	0.791	**0.958**
		MLE	**0.96**	1.931	0.968	1.729	0.968	1.638	0.96	1.607
		MCMC	0.95	2.528	0.95	2.038	0.95	1.81	0.95	1.792
	*β*_1_	Laplace	0.919	1.945	**0.944**	1.979	**0.947**	1.969	**0.95**	2.01
		Tangent	0.623	**1.052**	0.654	**1.058**	0.71	**1.055**	0.67	**1.06**
		MLE	**0.96**	2.828	0.963	2.482	0.958	2.292	0.954	2.225
		MCMC	0.95	3.489	0.95	3.032	0.95	2.71	0.95	2.584

The medians are calculated across the different occupancy and detection probability combinations for fixed values of *n* and *K*. *n* represents the total number of sites visited while *K* represents the number of visits to each site. We highlight the method with the smallest credibility/confidence interval width as well the method (not considering the MCMC method) with the closest coverage probability to 0.95.

We found that as the number of sites increased, the credibility (and confidence) interval widths of the true regression parameters decreased (for all methods). For a fixed number of sites, the credibility (and confidence) interval widths of the true ***α*** parameter values decreased as *K* increased while the associated widths for the ***β*** parameters did not appear to decrease noticeably with an increase in *K*. The simulation results suggests that the coverage probabilities associated with the Laplace method and the MLE methods (for all regression parameters) are very close to that of the nominal coverage value of 0.95 for *K* ≥ 3. It is evident from these results that the Tangent based method does not perform well under any of the scenarios considered and consistently produced the smallest credibility interval widths.

**Fig 1:**
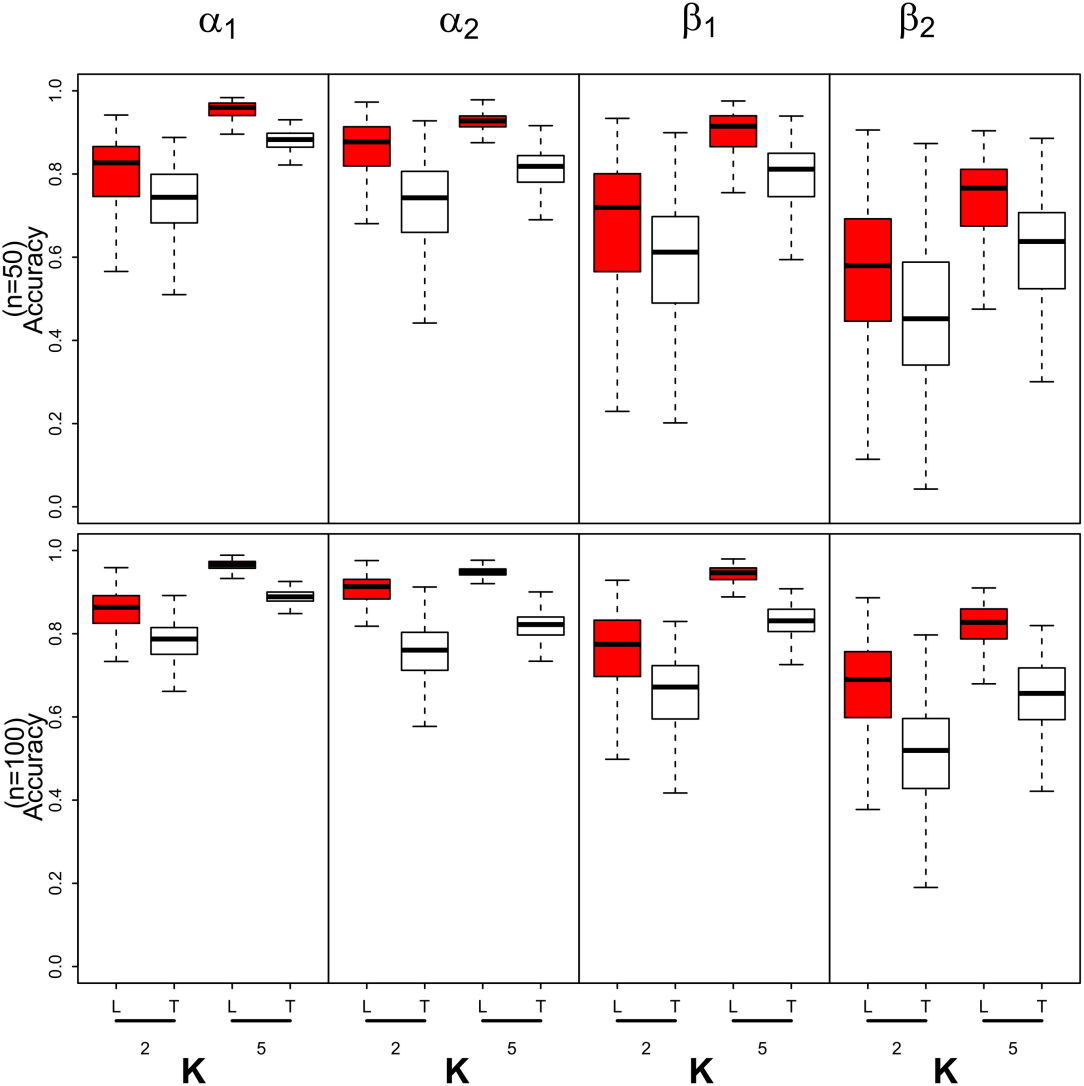
Box plots of the accuracy measurements for the model parameters associated with the Laplace (L-dark boxes) and Tangent (T-light boxes) based method for number of sites *n* = 50, 100 and number of visits to each site *K* = 2, 5. The detection and occupancy probabilities are approximately 0:5. The accuracy of the VB approximations is measured by calculating $\text{acc}(x)=1-\frac{1}{2}\int \left|q(x)-q_{MCMC}(x)\right|\;dx.$ The measure lies between 0 and 1 with a value of 1 indicating a perfect approximation and a value close to 0 indicating a poor approximation by the variational distribution to the true posterior distribution.

Based on the accuracy calculations across the replicate data sets, the Tangent based method generally appears to be worst at approximating the marginal posterior distributions of both ***α*** and ***β*** when comparisons are made based on the median accuracy measure for these parameters. As an example of these simulation results, consider the scenario where the estimated mean detection and occupancy probability across all sites and revisits are both 0.5 (see Fig 1). In general, the posterior approximations for the detection regression parameters were quite good with median accuracy statistics greater than 0.8 even when the number of revisits are small. The accuracy statistics for the detection regression parameters dramatically increase as *n* and *K* increases with median accuracy statistics in excess of 0.95 when *K* = 5. Similar comments can be made regarding the accuracy of the posterior approximations for the occupancy regression parameters. In general, the accuracies increase with an increase in *n* and *K* however the rate of increase in the accuracy statistics for the occupancy regression parameters appears slower than those observed for the detection regression parameters. The box plots of the accuracy statistics associated with the different methods for the remaining cases can be found in the Supporting Information (see S1, S2 and S3 Figs).

**Fig 2:**
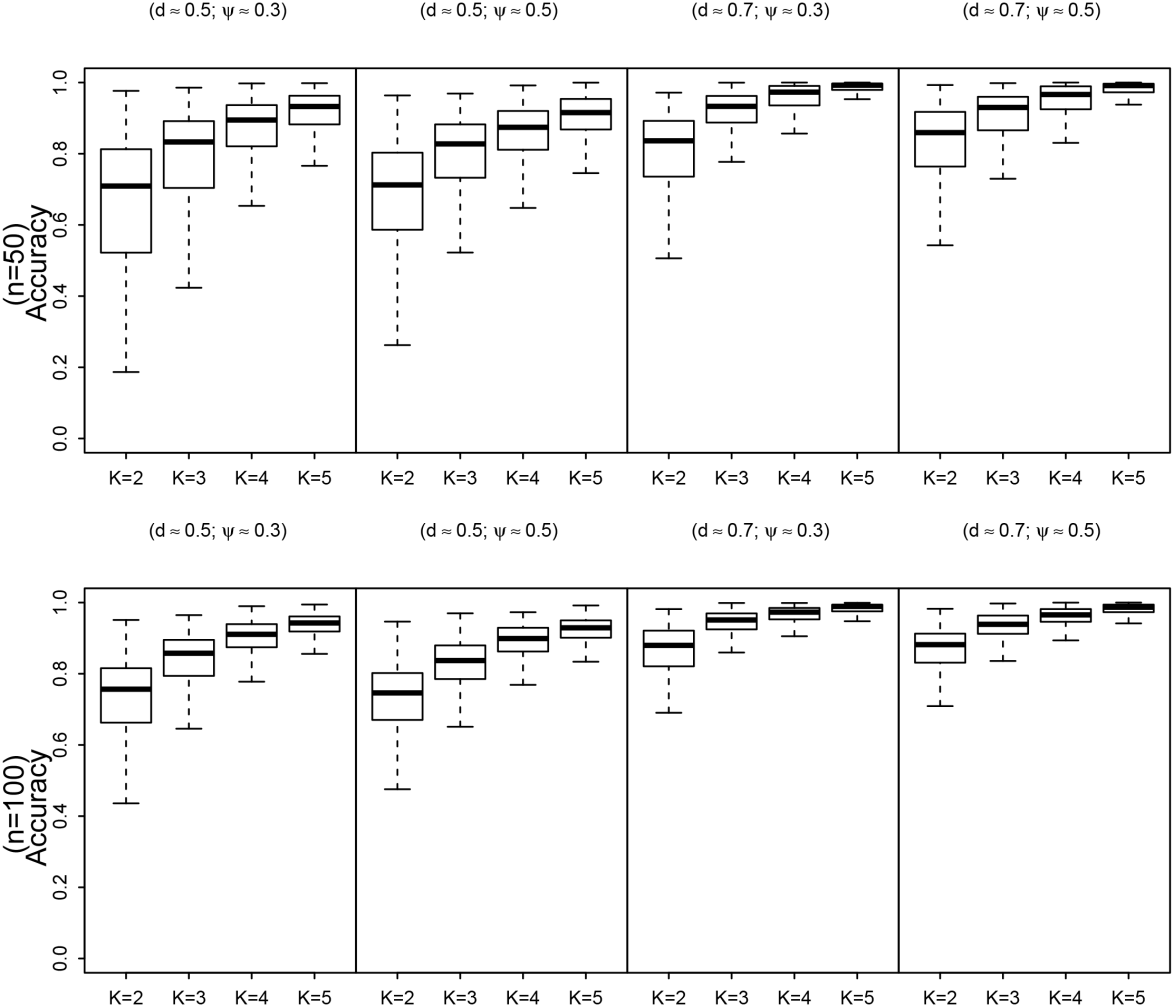
Box plots of the accuracy measurements for the predictive distribution of the proportions of occupied sites associated with the Laplace method for number of sites *n* = 50, 100 and number of visits to each site *K* = 2; 3; 4 and 5. The accuracy of the VB approximations is measured by calculating $\text{acc}(x)=1-\frac{1}{2}\int \left|q(x)-q_{MCMC}(x)\right|\;dx.$ The measure lies between 0 and 1 with a value of 1 indicating a perfect approximation and a value close to 0 indicating a poor approximation by the variational distribution to the true posterior distribution.

We found that the accuracy of the approximate predictive distributions for the proportion of occupied sites improves as *K* increases (see Fig 2). This observation is consistent across all of the scenarios considered. From an examination of the posterior predictive distributions (not shown here) it is evident that the VB predictive distributions are lighter tailed than the MCMC predictive distributions however this effect is reduced for *K* ≥ 3. This observation can clearly be seen when examining the results displayed in Tables 2 and 3. It is noticeable that the summary statistics of the predictive distributions using the two methods are very similar although the VB predictive distributions display a slightly reduced posterior variance under certain conditions.

**Table 2:** Summary statistics of the posterior predictive distributions of the proportion of sites occupied using the MCMC method and the VB method for different scenarios. (*n* = 50.).

		MCMC Method					Laplace Method				
Case	K	Mean	Std	Median	2.5%	97.5%	Mean	Std	Median	2.5%	97.5%
*d* ≈ 0.5 *ψ* ≈ 0.3	2	16.8	5.2	16	8	29	14.6	3.3	15	8	21
	3	15.6	3.6	15	10	24	14.6	2.9	14	9	20
	4	15.0	2.8	15	10	21	14.6	2.6	15	10	20
	5	14.9	2.6	15	10	20	14.7	2.5	15	10	20
*d* ≈ 0.7 *ψ* ≈ 0.3	2	25.7	4.7	25	18	36	23.9	3.5	24	17	31
	3	25.0	3.6	25	18	33	24.1	3.1	24	18	30
	4	25.0	3.0	25	19	31	24.6	2.8	25	19	30
	5	24.7	2.8	25	20	30	24.5	2.7	24	19	30
*d* ≈ 0.5 *ψ* ≈ 0.5	2	15.8	3.5	15	10	24	14.9	2.7	15	10	20
	3	14.8	2.5	15	10	20	14.5	2.4	14	10	20
	4	14.5	2.4	14	10	19	14.4	2.4	14	10	19
	5	14.6	2.4	15	10	19	14.6	2.4	15	10	19
*d* ≈ 0.7 *ψ* ≈ 0.5	2	25.2	3.3	25	19	32	24.6	2.9	25	19	30
	3	24.6	2.8	25	20	30	24.4	2.7	24	19	30
	4	24.6	2.5	25	20	30	24.6	2.5	25	20	29
	5	24.7	2.5	25	20	29	24.6	2.5	25	20	29

**Table 3:** Summary statistics of the posterior predictive distributions of the proportion of sites occupied using the MCMC method and the VB method for different scenarios. (*n* = 100.).

		MCMC Method					Laplace Method				
Case	K	Mean	Std	Median	2.5%	97.5%	Mean	Std	Median	2.5%	97.5%
*d* ≈ 0.5 *ψ* ≈ 0.3	2	31.0	6.3	30	21	45	29.4	4.6	29	21	39
	3	30.4	4.6	30	22	40	29.7	4.1	30	22	37
	4	29.7	3.8	30	22	37	29.4	3.6	29	22	36
	5	29.7	3.6	30	22	37	29.5	3.5	29	22	36
*d* ≈ 0.7 *ψ* ≈ 0.3	2	49.7	6.3	49	38	63	48.3	5.2	48	38	58
	3	49.6	4.9	50	40	59	48.9	4.6	49	40	58
	4	49.3	4.2	49	41	58	48.9	4.1	49	41	57
	5	49.1	3.9	49	41	57	48.9	3.9	49	41	56
*d* ≈ 0.5 *ψ* ≈ 0.5	2	30.2	4.4	30	22	40	29.7	4.0	30	22	38
	3	29.6	3.5	30	23	36	29.5	3.4	30	23	36
	4	29.5	3.3	30	23	36	29.4	3.3	29	23	36
	5	29.5	3.3	29	23	36	29.5	3.2	29	23	36
*d* ≈ 0.7 *ψ* ≈ 0.5	2	49.4	4.5	49	41	58	48.9	4.2	49	41	57
	3	49.2	3.8	49	42	57	49.0	3.8	49	42	56
	4	49.1	3.7	49	42	56	49.0	3.7	49	42	56
	5	49.0	3.7	49	41	56	49.0	3.7	49	41	56

Box plots of the accuracy measurements for the predictive distribution of the proportions of occupied sites associated with the Laplace method for number of sites *n* = 50, 100 and number of visits to each site *K* = 2, 3, 4 and 5. The accuracy of the VB approximations is measured by calculating $\text{acc}\left(x\right)=1-\frac{1}{2}\int \left|q\left(x\right)-q_{MCMC}\left(x\right)\right|dx$. The measure lies between 0 and 1 with a value of 1 indicating a perfect approximation and a value close to 0 indicating a poor approximation by the variational distribution to the true posterior distribution.

### 2.2 Application to real data sets

As examples of the proposed technique, we use detection-nondetection data extracted from the second Southern African Bird Atlas Project [31] database (see http://sabap2.adu.org.za/) for 2012 to compare the performance of different methods for fitting a single season occupancy model. The data were collected by citizen scientists using 5-minute latitude × 5-minute longitude rectangular grids across South Africa [31]. Each site is approximately 8 km × 7.6 km [8]. The citizen scientists were asked to make a list of all the species that they encountered during at least two hours of intense birding. They were allowed to add additional species to the list for up to five days. By providing information on the species that they encountered, the citizen scientists implicitly also provided information about the species they did not encounter. Hence, we extracted detection-nondetection data for five bird species (1. Black-headed heron (*Ardea melanocephala*), 2. Egyptian goose (*Alopochen aegyptiaca*), 3. orange-throated longclaw (*Macronyx capensis*), 4. white-browed sparrow-weaver (*Plocepasser mahali*) and 5. Long-tailed widowbird (*Euplectes progne*)) from this database, treating each check-list as an independent observation. We included all grid cells in and around Gauteng, South Africa, that contained a minimum of three site visits. Many of the sites were visited a large number of times but we limited the maximum number of site visits to five (since the focus of the analysis was to assess whether the VB techniques could be used to analyse studies which have relatively small sample sizes and low number of revisits per site). This restriction reduced the data sets to 123 sites; 50 of which had three surveys; 52 had four surveys and the remaining 21 sites had 5 surveys.

In our analysis we specifically compare the MLE, MCMC and the VB methods where uninformative priors (as in the simulation study) were used for all parameters. We fitted a model with one detection covariate and one occupancy covariate. The detection covariate used was the number of species observed by the birder (denoted as *nspp*) while the occupancy probability was modelled as a function of the ratio of potential to realized evapotranspiration (*AETdivPETs*). *AETdivPETs* is a measure of vegetation cover and hydric stress and is an important predictor for bird species occurrence in South Africa [32]. Both covariates were standardized to have zero mean and unit variance.

Maximum likelihood estimation was undertaken using the R package *unmarked*[33]; MCMC sampling was undertaken using the R package jagsUI [34] while all variational approximations were performed using the authors’ code. The R code used to perform the analysis (S1 Code), the data (S1 Data) as well as documentation regarding the VB code (S2 Code, S2 Data) can be found in the Supporting information. The MCMC estimation was undertaken as per the simulation study discussed previously.

**Fig 3:**
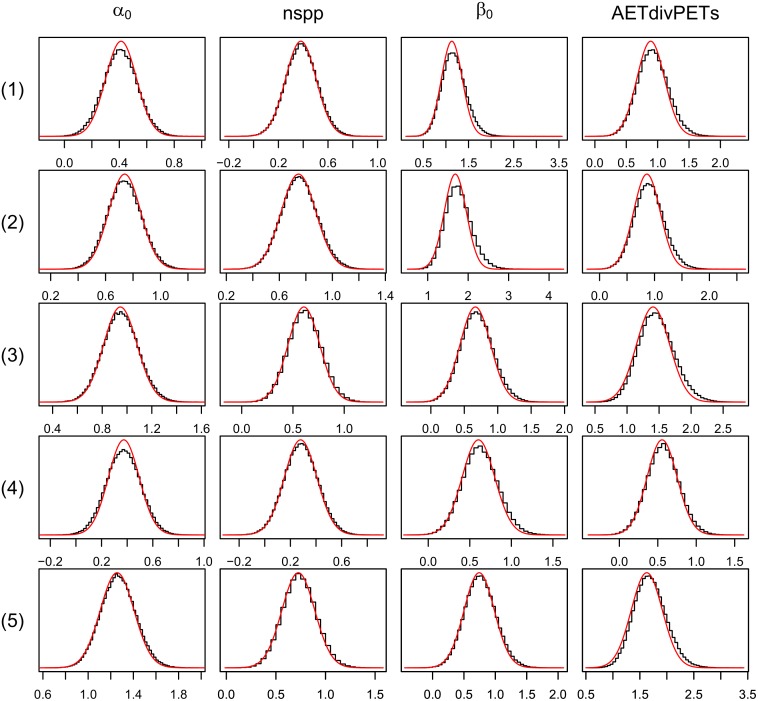
A comparison between the VB distributions (solid line) and the posterior distributions obtained using MCMC (the histogram) for the regression parameters of the detection and occupancy process for the different bird species (denoted as (1) = Black-headed heron, (2) = Egyptian goose, (3) = Orange-throated longclaw, (4) = White-browed sparrow-weaver and (5) = Long-tailed widowbird).

**Fig 4:**
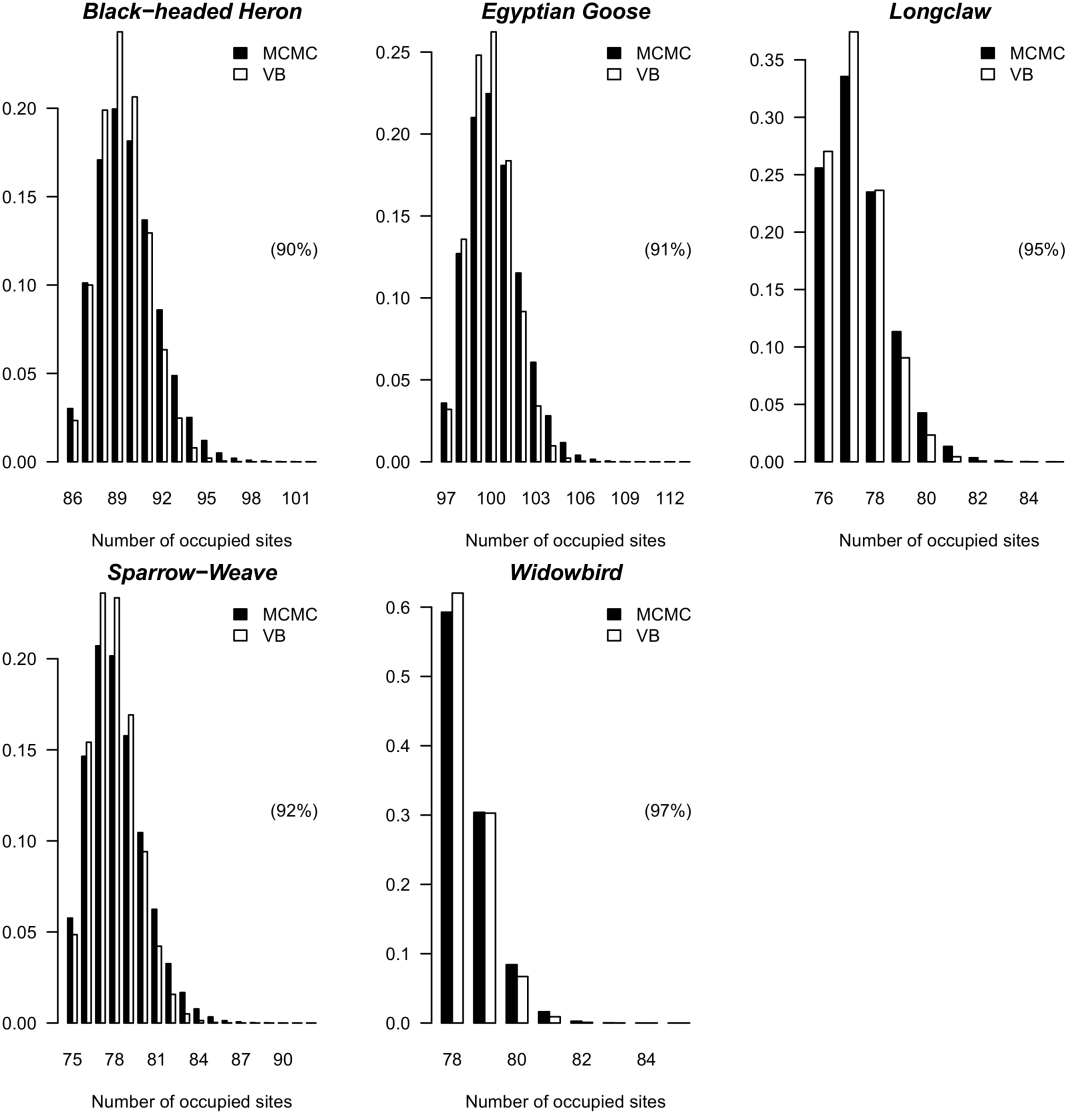
Predictive distribution of the proportions of occupied sites using the VB Laplace method and the MCMC method for the different bird species. The accuracy statistics (*acc*(*x*)) are displayed in brackets. The *acc*(*x*) measure lies between 0 and 1 with a value of 1 indicating a perfect approximation and a value close to 0 indicating a poor approximation by the variational distribution to the true posterior distribution.

The approximate posterior means and standard deviations of the VB distributions were all close to the posterior means and standard deviations obtained using MCMC (see Table 4 and Fig 3). The regression coefficients are all positive and statistically significantly different from zero. From an examination of the predictive distributions of the PAO for the different species it is evident that the VB distributions can be used to obtain accurate approximations to the true predictive distributions of the PAO (see Fig 4 and Table 5). Notice that the accuracy statistics for all of the species considered were above 0.9.

**Table 4:** Parameter estimates of the single season occupancy models fitted using MLE, VB and MCMC.

		MLE		VB		MCMC		
Species	Covariate	Est	SE	Mean	Std	Mean	Std	MCSE
Black headed Heron	Int (detection) nspp	0.412	0.123	0.413	0.113	0.408	0.124	<0.001
		0.381	0.128	0.381	0.123	0.386	0.128	<0.001
	Int (Occupancy) AETdivPETs	1.136	0.255	1.131	0.231	1.182	0.269	0.002
		0.892	0.236	0.890	0.221	0.926	0.246	0.002
Egyptian Goose	Int (detection) nspp	0.738	0.124	0.739	0.117	0.737	0.125	<0.001
		0.745	0.135	0.745	0.131	0.751	0.135	<0.001
	Int (Occupancy) AETdivPETs	1.690	0.300	1.682	0.273	1.764	0.323	0.003
		0.866	0.258	0.861	0.242	0.906	0.273	0.002
Orange throated longclaw	Int (detection) nspp	0.946	0.141	0.946	0.135	0.950	0.143	0.001
		0.607	0.159	0.607	0.154	0.618	0.160	0.001
	Int (Occupancy) AETdivPETs	0.668	0.238	0.665	0.229	0.690	0.243	0.001
		1.410	0.268	1.406	0.258	1.462	0.279	0.002
White browed sparrow weaver	Int (detection) nspp	0.372	0.132	0.374	0.120	0.370	0.133	0.001
		0.280	0.132	0.280	0.127	0.284	0.132	<0.001
	Int (Occupancy) AETdivPETs	0.607	0.210	0.604	0.197	0.626	0.213	0.001
		0.556	0.205	0.556	0.196	0.569	0.210	0.001
Long tailed widow bird	Int (detection) nspp	1.257	0.153	1.256	0.149	1.268	0.154	0.001
		0.727	0.168	0.726	0.166	0.738	0.169	0.001
	Int (Occupancy) AETdivPETs	0.746	0.245	0.743	0.241	0.763	0.251	0.002
		1.633	0.293	1.628	0.288	1.693	0.302	0.002

The estimation results indicate that the VB method accurately estimates the posterior means of all of the parameters while the posterior variances are marginally underestimated.

**Table 5:** Summary statistics of the posterior predictive distributions of the proportion of sites occupied using the MCMC method and the VB method for the five bird species considered.

Species	Method	Mean	Std	2.5%	97.5%
Black-headed	MCMC	89.7	2.05	86	94
Heron	VB	89.3	1.64	87	93
Egyptian	MCMC	100.2	1.78	97	104
goose	VB	99.9	1.47	97	103
Orange-throated	MCMC	77.4	1.23	76	80
longclaw	VB	77.2	1.05	76	80
White-browed	MCMC	78.2	1.99	75	83
sparrow-weaver	VB	77.9	1.63	75	81
Long-tailed	MCMC	78.5	0.74	78	80
widowbird	VB	78.5	0.67	78	80

## 3 Discussion

We developed two new methods of approximating the posterior distribution of the parameters of a Bayesian single season occupancy model that use logistic link functions. The first method uses a Laplace approximation of the VB optimal distributions while the second method utilizes the tangent based method of [35]. Based on the simulation studies it was found that the Laplace approximation method performed well under most conditions considered. We believe that the approximation results obtained using the probit link function would be similar to those obtained using the tangent based method and thus did not explicitly consider this link function here. The methods have laid the groundwork that would enable VB methods to be applied to more complicated occupancy models and are currently the focus of ongoing research.

One big advantage of the methods developed here is the fact that they could be applied to cases where the researcher has informative prior information and might not want to rely on the use of the MLE method. In that situation, Markov Chain Monte Carlo (MCMC) methods were so far the only methods available for fitting occupancy models in a Bayesian analysis. However, for big data sets, MCMC methods can be too slow to be useful. The code used to implement the methods is available in R and was at least 100 times faster than running MCMC using jagsUI **in our example**.

Simulations showed that when uninformative prior distributions were used, in general, the Laplace method attains very similar frequentist coverage probabilities to those obtained by the MLE method when the number of sampling occasions is at least three. We advise that the approximate methods could be used when the detection probability is at least 0.5 and there are at least three sampling occasions.

A further advantage of the methods developed here is the ease with which one can approximate the predictive distribution of the proportion of area occupied. Our simulation results showed that the Laplace approximate method can be used to obtain approximate distributions of the PAO. For scenarios where the detection probabilities are relatively low and the number of sites visits are small (*K* = 2) we found that the approximate methods slightly under estimate the upper bound of the PAO. The differences between the true predictive distribution and the approximate one is however very small for *K* ≥ 3.

In both of the methods considered the approximate distributions derived are both multivariate Gaussian. When the sample size is particularly small, the number of sampling occasions is low (possibly one or two) or when the detection probability is low (less than 0.3) we have found that the posterior distributions of the parameters of the model are often skewed (particularly the occupancy covariate parameters). In these cases the approximate methods do not work well. Future work could entail the use of skew distributions similar to that proposed by [36].

## Supporting Information

S1 Text Some notation and distribution theory used in the main part of the text. (PDF)

S1 Appendix Derivation of the lower bound to the joint likelihood and the VB distributions. (PDF)

S2 Appendix Derivation of the tangent based method. (PDF)

S3 Appendix Explanation regarding the convergence calculations. (PDF)

S1 Fig Box plots of the accuracy measurements for the model parameters associated with the Laplace (L-dark boxes) and Tangent (T-light boxes) based method for number of sites *n* = 50, 100 and number of visits to each site *K* = 2, 5. The detection probability is approximately 0.5 while the occupancy probability is approximately 0.3.
(TIF)

S2 Fig Box plots of the accuracy measurements for the model parameters associated with the Laplace (L-dark boxes) and Tangent (T-light boxes) based method for number of sites *n* = 50, 100 and number of visits to each site *K* = 2, 5. The detection probability is approximately 0.7 while the occupancy probability is approximately 0.3.
(TIF)

S3 Fig Box plots of the accuracy measurements for the model parameters associated with the Laplace (L-dark boxes) and Tangent (T-light boxes) based method for number of sites *n* = 50, 100 and number of visits to each site *K* = 2, 5. The detection probability is approximately 0.7 while the occupancy probability is approximately 0.5.
(TIF)

S1 Code The R code used to undertake the analysis. (R)

S2 Code How to use the VB Laplace approximation code. (PDF)

S1 Data The R data file that contains the data used to undertake the analysis. (RDATA)

S2 Data The R data file that contains the data used to explain how to use the VB Laplace approximation code. (RDA)

## References

[1] Robert C, Casella G. Monte Carlo statistical methods. Springer Science & Business Media; 2013.

[2] Kuhnert PM, Martin TG, Griffiths SP. A guide to eliciting and using expert knowledge in Bayesian ecological models. Ecology Letters. 2010;13(7):900–914. 10.1111/j.1461-0248.2010.01477.x 20497209

[3] Choy SL, O’Leary R, Mengersen K. Elicitation by design in ecology: using expert opinion to inform priors for Bayesian statistical models. Ecology. 2009;90(1):265–277. 10.1890/07-1886.1 19294931

[4] Clark JS. Why environmental scientists are becoming Bayesians. Ecology letters. 2005;8(1):2–14. 10.1111/j.1461-0248.2004.00702.x

[5] MacKenzie DI, Nichols JD, Lachman GB, Droege S, Royle JA, Langtimm CA. Estimating site occupancy rates when detection probabilities are less than one. Ecology. 2002;83(8):2248–2255. 10.1890/0012-9658(2002)083[2248:ESORWD]2.0.CO;2

[6] Bailey LL, MacKenzie DI, Nichols JD. Advances and applications of occupancy models. Methods in Ecology and Evolution. 2014;5(12):1269–1279. 10.1111/2041-210X.12100

[7] Bled F, Nichols JD, Altwegg R. Dynamic occupancy models for analyzing species’ range dynamics across large geographic scales. Ecology and Evolution. 2013;3(15):4896–4909. 10.1002/ece3.858 24455124 PMC3892356

[8] Broms KM, Johnson DS, Altwegg R, Conquest LL. Spatial occupancy models applied to atlas data show Southern Ground Hornbills strongly depend on protected areas. Ecological Applications. 2014;24(2):363–374. 10.1890/12-2151.1 24689147

[9] Royle JA, Dorazio RM. Hierarchical modeling and inference in ecology: the analysis of data from populations, metapopulations and communities. Academic Press, San Diego, CA; 2008.

[10] Johnson DS, Conn PB, Hooten MB, Ray JC, Pond BA. Spatial occupancy models for large data sets. Ecology. 2013;94(4):801–808. 10.1890/12-0564.1

[11] Royle JA, Kery M. A Bayesian state-space formulation of dynamic occupancy models. Ecology. 2007;88(7):1813–1823. 10.1890/06-0669.1 17645027

[12] Welsh AH, Lindenmayer DB, Donnelly CF. Fitting and interpreting occupancy models. PLoS One. 2013;8(1):e52015 10.1371/journal.pone.0052015 23326323 PMC3542396

[13] Guillera-Arroita G, Lahoz-Monfort JJ, MacKenzie DI, Wintle BA, McCarthy MA. Ignoring Imperfect Detection in Biological Surveys Is Dangerous: A Response to’Fitting and Interpreting Occupancy Models’. PloS One. 2014;9(7):e99571 10.1371/journal.pone.0099571 25075615 PMC4116132

[14] Hutchinson RA, Valente JJ, Emerson SC, Betts MG, Dietterich TG. Penalized likelihood methods improve parameter estimates in occupancy models. Methods in Ecology and Evolution. 2015;6(8):949–959. 10.1111/2041-210X.12368

[15] Moreno M, Lele SR. Improved estimation of site occupancy using penalized likelihood. Ecology. 2010;91(2):341–346. 10.1890/09-1073.1 20391998

[16] MacKenzie DI. Occupancy estimation and modeling: inferring patterns and dynamics of species occurrence. Academic Press; 2006.

[17] Kéry M, Gardner B, Monnerat C. Predicting species distributions from checklist data using site-occupancy models. Journal of Biogeography. 2010;37(10):1851–1862.

[18] Kullback S, Leibler RA. On information and sufficiency. The Annals of mathematical statistics. 1951;22(1):79–86. 10.1214/aoms/1177729694

[19] Ormerod JT, Wand MP. Explaining variational approximations. The American Statistician. 2010;62(2):140–153. 10.1198/tast.2010.09058

[20] McGrory CA, Titterington D. Variational approximations in Bayesian model selection for finite mixture distributions. Computational Statistics & Data Analysis. 2007;51(11):5352–5367. 10.1016/j.csda.2006.07.020

[21] Bishop CM. A new framework for machine learning In: Computational Intelligence: Research Frontiers. Springer; 2008 p. 1–24.

[22] Hensman J, Rattray M, Lawrence ND. Fast variational inference in the conjugate exponential family. In: Advances in Neural Information Processing Systems; 2012 p. 2888–2896.

[23] Wang B, Titterington DM. Inadequacy of interval estimates corresponding to variational Bayesian approximations. Proceedings of the 10th International Workshop on Artificial Intelligence; 2005.

[24] Grimmer J. An introduction to Bayesian inference via variational approximations. Political Analysis. 2010;19(1):32–47. 10.1093/pan/mpq027

[25] You C, Ormerod JT, Müller S. On variational Bayes estimation and variational information criteria for linear regression models. Australian and New Zealand Journal of Statistics. 2014;56(1):73–87. 10.1111/anzs.12063

[26] Nathoo FS, Babul A, Moiseev A, Virji-Babul N, Beg MF. A variational Bayes spatiotemporal model for electromagnetic brain mapping. Biometrics. 2014;70(1):132–143. 10.1111/biom.12126 24354514

[27] R Core Team. R: A Language and Environment for Statistical Computing; 2014. Available from: http://www.R-project.org/.

[28] Su, YS, Yajima, M. R2jags: A Package for Running jags from R. R package version 003-08, URL http://CRAN.R-project org/package=R2jags. 2012;.

[29] Plummer M. JAGS: A program for analysis of Bayesian graphical models using Gibbs sampling. In: Proceedings of the 3rd international workshop on distributed statistical computing. vol. 124. Technische Universit at Wien Wien, Austria; 2003. p. 125.

[30] Link WA, Eaton MJ. On thinning of chains in MCMC. Methods in Ecology and Evolution. 2012;3(1):112–115. 10.1111/j.2041-210X.2011.00131.x

[31] Harebottle DM, Smith N, Underhill LG, Brooks M. The Southern African Bird Atlas Project 2; 2007.

[32] Péron G, Altwegg R. The abundant centre syndrome and species distributions: insights from closely related species pairs in southern Africa. Global Ecology and Biogeography. 2015;24(2):215–225. 10.1111/geb.12251

[33] Fiske I, Chandler R. unmarked: An R package for fitting hierarchical models of wildlife occurrence and abundance. Journal of Statistical Software. 2011;43(10):1–23. 10.18637/jss.v043.i10

[34] Kellner K. jagsUI: Run JAGS (specifically, libjags) from R; an alternative user interface for rjags. R package version. 2014;1.

[35] Jaakkola TS, Jordan MI. Bayesian logistic regression: a variational approach. Statistics and Computing. 2000;10(2):25–37. 10.1023/A:1008932416310

[36] Ormerod JT. Skew-Normal Variational Approximations for Bayesian Inference. School of Mathematics and Statistics, University of Sydney, Technical Report CRG- TR-93-1; 2011.

