# Supplementary material for: A Variational Bayes Approach to the Analysis of Occupancy Models
Source: PLoS One. 2016 Feb 29;11(2):e0148966. doi: 10.1371/journal.pone.0148966 (PMC4771718; doi:10.1371/journal.pone.0148966)
Supplement: S1 Text — (PDF) [file pone.0148966.s001.pdf]

S1 Text

Some matrix notation used in text.

Below we list some notation and distribution theory used in the main part of the text.

If $\boldsymbol{A}$ is the $b \times b$ matrix with entries denoted as a_{ij} $i = 1, \ldots, b,\ j = 1, \ldots, b$, $\boldsymbol{B} = \text{diag}(\boldsymbol{A})$ is the diagonal matrix with diagonal elements $b_{ii} = a_{ii}$ and off-diagonal elements all equal to zero. The identity diagonal($\boldsymbol{A}$) denotes the $b \times 1$ vector containing the diagonal elements of the matrix $\boldsymbol{A}$.

The trace of a matrix is defined as the sum of the diagonal elements of a matrix such that $\text{tr}(\boldsymbol{A}) = \sum_i a_{ii}$.

The transpose of the matrix $\boldsymbol{A}$ is denoted as $\boldsymbol{A}^T$.

Define $b(x)$ as some real valued function such that $b'(x)$ and $b''(x)$ are the 1st and 2nd derivative of $b(x)$ respectively. Let $\boldsymbol{l}$ be a column vector such that $b\left(\boldsymbol{l}\right)$ is a column vector where the ith element is $b(l_i)$.

$\boldsymbol{A} \odot \boldsymbol{B}$ denotes the Hadamard product. This operation performs element-wise multiplication of the elements in $\boldsymbol{A}$ and $\boldsymbol{B}$ where $\boldsymbol{A}$ and $\boldsymbol{B}$ are conformable matrices.

If $\boldsymbol{X}_p \sim N(\boldsymbol{\mu}\ \boldsymbol{\Sigma})$ then for some matrix $\boldsymbol{\Gamma}$, $E(\boldsymbol{X}^T\boldsymbol{\Gamma}\boldsymbol{X}) = \text{tr}\left(\boldsymbol{\Gamma}\boldsymbol{\Sigma}\right) + \boldsymbol{\mu}^T\boldsymbol{\Gamma}\boldsymbol{\mu}$. The entropy of the Gaussian distribution is defined as $\int f(\boldsymbol{x}) \ln f(\boldsymbol{x}) d\boldsymbol{x} = -\frac{p}{2}\left(\ln(2\pi) + 1\right) - \frac{1}{2}\log|\boldsymbol{\Sigma}|$.
