# Supplementary material for: A Variational Bayes Approach to the Analysis of Occupancy Models
Source: PLoS One. 2016 Feb 29;11(2):e0148966. doi: 10.1371/journal.pone.0148966 (PMC4771718; doi:10.1371/journal.pone.0148966)
Supplement: S1 Appendix — (PDF) [file pone.0148966.s002.pdf]

S2 Appendix

Lower bound to the joint likelihood and the VB distributions.

If we assume that the prior distribution for $\boldsymbol{\alpha}$ and $\boldsymbol{\beta}$ are multivariate Gaussian distributions (denoted as $\pi(\boldsymbol{\alpha}, \boldsymbol{\beta})$) with parameters $\boldsymbol{\mu}^0_{\boldsymbol{\alpha}}$, $\boldsymbol{\Sigma}^0_{\boldsymbol{\alpha}}$ and $\boldsymbol{\mu}^0_{\boldsymbol{\beta}}$, $\boldsymbol{\Sigma}^0_{\boldsymbol{\beta}}$ respectively then;

$$p(\boldsymbol{y}, \boldsymbol{z}, \boldsymbol{\alpha}, \boldsymbol{\beta}) = \pi(\boldsymbol{\alpha}, \boldsymbol{\beta}) \prod_{i=1}^{n} o_i^{z_i} (1 - o_i)^{1-z_i} \prod_{j=1}^{K_j} (z_i d_{i,j})^{y_{i,j}} (1 - z_i d_{i,j})^{1-y_{i,j}}$$

where we assume that we visit n sites $\boldsymbol{K} = (K_1, K_2, \ldots, K_n)^T$ times respectively. Let $b(x) = \ln(1 + \exp(x))$. $\ln \underline{p} = \ln p(\boldsymbol{y}, \boldsymbol{z}, \boldsymbol{\alpha}, \boldsymbol{\beta})$ is equal to

$$\begin{aligned} \ln \underline{p} &= \sum_{\{i:z_i=1\}} \sum_{j=1}^{K_j} \left(y_{i,j} \boldsymbol{w}_{i,j} \boldsymbol{\alpha} - b\left(\boldsymbol{w}_{i,j} \boldsymbol{\alpha}\right)\right) + \boldsymbol{z}^T \boldsymbol{X} \boldsymbol{\beta} - \mathbf{1}_n^T b\left(\boldsymbol{X}\boldsymbol{\beta}\right) + \ln \pi(\boldsymbol{\alpha}, \boldsymbol{\beta}) \\ &= \boldsymbol{y}^T \mathrm{diag}(\tilde{\boldsymbol{z}}) \boldsymbol{W} \boldsymbol{\alpha} - \tilde{\boldsymbol{z}}^T b\left(\boldsymbol{W}\boldsymbol{\alpha}\right) + \boldsymbol{z}^T \boldsymbol{X} \boldsymbol{\beta} - \mathbf{1}_n^T b\left(\boldsymbol{X}\boldsymbol{\beta}\right) + \ln \pi(\boldsymbol{\alpha}, \boldsymbol{\beta}). \end{aligned} \quad (1)$$

Equation (1) follows after some simplification and since when $z_i = 0$ we have $\boldsymbol{y}_i = \mathbf{0}$. Here $\tilde{\boldsymbol{z}} = (z_1 \mathbf{1}_{K_1}^T, \ldots, z_n \mathbf{1}_{K_n}^T)^T$.

A lower bound on the marginal log-likelihood can be obtained using

$$\ln p(\boldsymbol{y}) \geq \sum_{\boldsymbol{z}} \int q(\boldsymbol{z}, \boldsymbol{\alpha}, \boldsymbol{\beta}) \ln \left(\frac{p(\boldsymbol{y}, \boldsymbol{z}, \boldsymbol{\alpha}, \boldsymbol{\beta})}{q(\boldsymbol{z}, \boldsymbol{\alpha}, \boldsymbol{\beta})} \right) d\boldsymbol{\alpha} d\boldsymbol{\beta}, \quad (2)$$

where $q(\boldsymbol{z}, \boldsymbol{\alpha}, \boldsymbol{\beta}) = q(\boldsymbol{z}) q(\boldsymbol{\alpha}) q(\boldsymbol{\beta})$. VB methods approximate the joint distribution of $\pi(\boldsymbol{z}, \boldsymbol{\alpha}, \boldsymbol{\beta} | \boldsymbol{y})$ by maximising the lower bound of the marginal log-likelihood and providing update equations for $q(\boldsymbol{z})$, $q(\boldsymbol{\alpha})$ and $q(\boldsymbol{\beta})$ in turn. Note that the dependence on $\boldsymbol{y}$ has been excluded from the notation for the variational distributions in order to save space.

Note that the variational posterior distribution of $\boldsymbol{\alpha}$ follows since

$$
\begin{aligned}
\ln q(\boldsymbol{\alpha}) &\propto \mathbb{E}_{\boldsymbol{\beta}}\left(\ln \underline{p}\right) \\
&\propto \mathbb{E}_{\boldsymbol{\beta}}\left(\boldsymbol{y}^T \mathrm{diag}(\tilde{\boldsymbol{z}})\boldsymbol{W}\boldsymbol{\alpha} - \tilde{\boldsymbol{z}}^T b\left(\boldsymbol{W}\boldsymbol{\alpha}\right) + \boldsymbol{z}^T\boldsymbol{X}\boldsymbol{\beta} - \mathbf{1}_n^T b\left(\boldsymbol{X}\boldsymbol{\beta}\right) + \ln \pi(\boldsymbol{\alpha}, \boldsymbol{\beta})\right) \\
&\propto \boldsymbol{y}^T \mathrm{diag}(\tilde{\boldsymbol{z}})\boldsymbol{W}\boldsymbol{\alpha} - \tilde{\boldsymbol{z}}^T b\left(\boldsymbol{W}\boldsymbol{\alpha}\right) + \ln \pi(\boldsymbol{\alpha}).
\end{aligned}
$$

Similarly

$$
\begin{aligned}
\ln q(\boldsymbol{\beta}) &\propto \mathbb{E}_{\boldsymbol{\alpha}}\left(\ln \underline{p}\right) \\
&\propto \mathbb{E}_{\boldsymbol{\alpha}}\left(\boldsymbol{y}^T \mathrm{diag}(\tilde{\boldsymbol{z}})\boldsymbol{W}\boldsymbol{\alpha} - \tilde{\boldsymbol{z}}^T b\left(\boldsymbol{W}\boldsymbol{\alpha}\right) + \boldsymbol{z}^T\boldsymbol{X}\boldsymbol{\beta} - \mathbf{1}_n^T b\left(\boldsymbol{X}\boldsymbol{\beta}\right) + \ln \pi(\boldsymbol{\alpha}, \boldsymbol{\beta})\right) \\
&\propto \boldsymbol{z}^T\boldsymbol{X}\boldsymbol{\beta} - \mathbf{1}_n^T b\left(\boldsymbol{X}\boldsymbol{\beta}\right) + \ln \pi(\boldsymbol{\beta}).
\end{aligned}
$$
