# Supplementary material for: A Variational Bayes Approach to the Analysis of Occupancy Models
Source: PLoS One. 2016 Feb 29;11(2):e0148966. doi: 10.1371/journal.pone.0148966 (PMC4771718; doi:10.1371/journal.pone.0148966)
Supplement: S2 Appendix — (PDF) [file pone.0148966.s003.pdf]

S3 Appendix

A tangent based method.

The integrations required to obtain the lower bound of the marginal log-likelihood are non-trivial and no closed form equations for $q(\boldsymbol{z}, \boldsymbol{\alpha}, \boldsymbol{\beta})$ exists. Additional variational parameters $\boldsymbol{v} = (\boldsymbol{a}^T, \boldsymbol{b}^T)^T$ are now introduced such that a lower bound of the marginal log-likelihood is found using

$$\ln p(\boldsymbol{y}) \geq \sum_{\boldsymbol{z}} \int q_{\boldsymbol{v}}(\boldsymbol{z}) q_{\boldsymbol{v}}(\boldsymbol{\alpha}) q_{\boldsymbol{v}}(\boldsymbol{\beta}) \ln \left(\frac{p_{\boldsymbol{v}}(\boldsymbol{y}, \boldsymbol{z}, \boldsymbol{\alpha}, \boldsymbol{\beta})}{q_{\boldsymbol{v}}(\boldsymbol{z}) q_{\boldsymbol{v}}(\boldsymbol{\alpha}) q_{\boldsymbol{v}}(\boldsymbol{\beta})} \right) d\boldsymbol{\alpha} d\boldsymbol{\beta}. \tag{1}$$

$\boldsymbol{a} = (\underline{\boldsymbol{a}}_1^T, \ldots, \underline{\boldsymbol{a}}_n^T)^T$ where each of the $\underline{\boldsymbol{a}}_i$ vectors are of length K_i while $\boldsymbol{b}$ is of length n. $p_{\boldsymbol{v}}(\boldsymbol{y}, \boldsymbol{z}, \boldsymbol{\alpha}, \boldsymbol{\beta})$ should be viewed as a lower bound for $p(\boldsymbol{y}, \boldsymbol{z}, \boldsymbol{\alpha}, \boldsymbol{\beta})$ while $q_{\boldsymbol{v}}(\boldsymbol{z})$, $q_{\boldsymbol{v}}(\boldsymbol{\alpha})$ and $q_{\boldsymbol{v}}(\boldsymbol{\beta})$ indicates that the distributions of $\boldsymbol{z}$, $\boldsymbol{\alpha}$ and $\boldsymbol{\beta}$ are functions of $\boldsymbol{v}$ which could have different functional forms.

The function $f(x) = -\ln(1 + e^x)$ can be presented as the maximum of a family of parabolas [1] where

$$\begin{aligned}
-\ln(1 + e^x) &= \max_{\xi \in \Re} \left(A(\xi) x^2 - \frac{1}{2} x + C(\xi) \right) \text{ for all } x \in \Re, \\
A(\xi) &= -\tanh\left(\frac{1}{2}\xi\right) / (4\xi), \\
C(\xi) &= \frac{1}{2}\xi - \ln(1 + e^{\xi}) + \xi \tanh\left(\frac{1}{2}\xi\right) / 4.
\end{aligned}$$

A lower bound for the quantities $b(\boldsymbol{W\alpha})$ and $b(\boldsymbol{X\beta})$ are obtained by using the above result such

that

$$
\begin{aligned}
\ln \underline{p} &\geq \boldsymbol{y}^T \mathrm{diag}(\tilde{\boldsymbol{z}})\boldsymbol{W}\boldsymbol{\alpha} + \boldsymbol{z}^T\boldsymbol{X}\boldsymbol{\beta} + \ln\pi(\boldsymbol{\alpha},\boldsymbol{\beta}) + \tilde{\boldsymbol{z}}^T\left(A(\boldsymbol{a})\odot(\boldsymbol{W}\boldsymbol{\alpha})^2 - \frac{1}{2}\boldsymbol{W}\boldsymbol{\alpha} + C(\boldsymbol{a})\right) \\
&\quad +\mathbf{1}_n^T\left(A(\boldsymbol{b})\odot(\boldsymbol{X}\boldsymbol{\beta})^2 - \frac{1}{2}\boldsymbol{X}\boldsymbol{\beta} + C(\boldsymbol{b})\right) \\
&\geq -\frac{1}{2}\boldsymbol{\alpha}^T\boldsymbol{B}_1\boldsymbol{\alpha} + \boldsymbol{B}_2\boldsymbol{\alpha} - \frac{1}{2}\beta^T\boldsymbol{D}_1\boldsymbol{\beta} + \boldsymbol{D}_2\boldsymbol{\beta} + \tilde{\boldsymbol{z}}^T C(\boldsymbol{a}) + \mathbf{1}_n^T C(\boldsymbol{b}) + E. \qquad (2)
\end{aligned}
$$

Note that

$$
\begin{aligned}
\tilde{\boldsymbol{z}}^T\left(A(\boldsymbol{a})\odot(\boldsymbol{W}\boldsymbol{\alpha})^2\right) &= \boldsymbol{\alpha}^T\boldsymbol{W}^T\mathrm{diag}(A(\boldsymbol{a})\odot\tilde{\boldsymbol{z}})\boldsymbol{W}\boldsymbol{\alpha} \\
1_n^T\left(A(\boldsymbol{b})\odot(\boldsymbol{X}\boldsymbol{\beta})^2\right) &= \boldsymbol{\beta}^T\boldsymbol{X}^T\mathrm{diag}(A(\boldsymbol{b}))\boldsymbol{X}\boldsymbol{\beta}
\end{aligned}
$$

with

$$
\begin{aligned}
\boldsymbol{B}_1 &= (\boldsymbol{\Sigma}^0_{\boldsymbol{\alpha}})^{-1} - 2\boldsymbol{W}^T\mathrm{diag}(A(\boldsymbol{a})\odot\tilde{\boldsymbol{p}})\boldsymbol{W} \\
\boldsymbol{B}_2 &= \left(\tilde{\boldsymbol{P}}\boldsymbol{y} - \frac{1}{2}\tilde{\boldsymbol{p}}\right)^T\boldsymbol{W} + (\boldsymbol{\mu}^0_{\boldsymbol{\alpha}})^T(\boldsymbol{\Sigma}^0_{\boldsymbol{\alpha}})^{-1} \\
\boldsymbol{D}_1 &= (\boldsymbol{\Sigma}^0_{\boldsymbol{\beta}})^{-1} - 2\boldsymbol{X}^T\mathrm{diag}(A(\boldsymbol{b}))\boldsymbol{X} \\
\boldsymbol{D}_2 &= \left(\boldsymbol{p} - \frac{1}{2}\mathbf{1}_n\right)^T\boldsymbol{X} + (\boldsymbol{\mu}^0_{\boldsymbol{\beta}})^T(\boldsymbol{\Sigma}^0_{\boldsymbol{\beta}})^{-1} \\
E &= -\frac{1}{2}\left(\ln 2\pi(p+q) + \ln|\boldsymbol{\Sigma}^0_{\boldsymbol{\alpha}}| + \ln|\boldsymbol{\Sigma}^0_{\boldsymbol{\beta}}| + (\boldsymbol{\mu}^0_{\boldsymbol{\alpha}})^T(\boldsymbol{\Sigma}^0_{\boldsymbol{\alpha}})^{-1}\boldsymbol{\mu}^0_{\boldsymbol{\alpha}} + (\boldsymbol{\mu}^0_{\boldsymbol{\beta}})^T(\boldsymbol{\Sigma}^0_{\boldsymbol{\beta}})^{-1}\boldsymbol{\mu}^0_{\boldsymbol{\beta}}\right).
\end{aligned}
$$

From equation (2) it is apparent that both $q_{\boldsymbol{v}}(\boldsymbol{\alpha})$ and $q_{\boldsymbol{v}}(\boldsymbol{\beta})$ are multivariate Gaussian distributions; specifically $q_{\boldsymbol{v}}(\boldsymbol{\alpha}) \sim N(\boldsymbol{B}_1^{-1}\boldsymbol{B}_2^T\ ,\ \boldsymbol{B}_1^{-1})$ and $q_{\boldsymbol{v}}(\boldsymbol{\beta}) \sim N(\boldsymbol{D}_1^{-1}\boldsymbol{D}_2^T\ ,\ \boldsymbol{D}_1^{-1})$. Using various known matrix

identities regarding multivariate Gaussian distributions it can be shown that

$$c_i^{(T)} = \boldsymbol{x}_i \boldsymbol{\mu}_{\boldsymbol{\beta}} + \mathbf{1}_{K_i}^T C(\underline{\boldsymbol{a}}_i) - \frac{1}{2}\mathbf{1}_{K_i}^T \underline{\boldsymbol{w}}_i \boldsymbol{\mu}_{\boldsymbol{\alpha}} + \operatorname{tr}(\boldsymbol{d}_i) \tag{3}$$

where $\boldsymbol{d}_i = \underline{\boldsymbol{w}}_i^T \mathrm{diag}(A(\underline{\boldsymbol{a}}_i))\underline{\boldsymbol{w}}_i \left(\boldsymbol{\Sigma}_{\boldsymbol{\alpha}} + \boldsymbol{\mu}_{\boldsymbol{\alpha}}\boldsymbol{\mu}_{\boldsymbol{\alpha}}^T\right)$. One way of estimating the variational parameters is to numerically maximise the right hand side of equation (1) with respect to $\boldsymbol{K}$. This approach might be feasible although it is not attempted here. Similar to [1] we however devise an Tangent algorithm in order to estimate $\boldsymbol{v}$.

Estimation of the variational parameters - The E-step

Denote the 'new variational parameters' as $\boldsymbol{a}_{(\boldsymbol{N})}$ and $\boldsymbol{b}_{(\boldsymbol{N})}$ respectively. Further denote $\boldsymbol{v}^{(new)}$ as $\boldsymbol{N}$ and $\boldsymbol{v}^{(old)}$ as $\boldsymbol{O}$ for notational convenience below. Treating $\boldsymbol{y}, \boldsymbol{z}, \boldsymbol{\alpha}, \boldsymbol{\beta}$ as the 'complete data', the E-step of the Tangent algorithm is found by calculating the conditional expectation of the right hand side of equation (2) which equals $Q(\boldsymbol{N}|\boldsymbol{O}) = T_{\boldsymbol{\alpha},\boldsymbol{N}} + T_{\boldsymbol{\beta},\boldsymbol{N}} + E$ where

$$T_{\boldsymbol{\alpha},\boldsymbol{N}} = \operatorname{tr}\left(-\frac{1}{2}\boldsymbol{B}_{1,\boldsymbol{N}}\left(\boldsymbol{\Sigma}_{\boldsymbol{\alpha},\boldsymbol{O}} + \boldsymbol{\mu}_{\boldsymbol{\alpha},\boldsymbol{O}}\boldsymbol{\mu}_{\boldsymbol{\alpha},\boldsymbol{O}}^T\right)\right) + \boldsymbol{B}_2\boldsymbol{\mu}_{\boldsymbol{\alpha},\boldsymbol{O}} + \boldsymbol{B}_{3,\boldsymbol{N}}$$
$$T_{\boldsymbol{\beta},\boldsymbol{N}} = \operatorname{tr}\left(-\frac{1}{2}\boldsymbol{D}_{1,\boldsymbol{N}}\left(\boldsymbol{\Sigma}_{\boldsymbol{\beta},\boldsymbol{O}} + \boldsymbol{\mu}_{\boldsymbol{\beta},\boldsymbol{O}}\boldsymbol{\mu}_{\boldsymbol{\beta},\boldsymbol{O}}^T\right)\right) + \boldsymbol{D}_2\boldsymbol{\mu}_{\boldsymbol{\beta},\boldsymbol{O}} + \boldsymbol{D}_{3,\boldsymbol{N}}.$$

where

$$\boldsymbol{B}_{3,N} = \tilde{\boldsymbol{p}}^T C(\boldsymbol{a}_{\boldsymbol{N}}) - \frac{1}{2}(\boldsymbol{\mu}_{\boldsymbol{\alpha}}^0)^T(\boldsymbol{\Sigma}_{\boldsymbol{\alpha}}^0)^{-1}(\boldsymbol{\mu}_{\boldsymbol{\alpha}}^0)$$
$$\boldsymbol{D}_{3,\boldsymbol{N}} = \mathbf{1}_n^T C(\boldsymbol{b}_{\boldsymbol{N}}) - \frac{1}{2}(\boldsymbol{\mu}_{\boldsymbol{\beta}}^0)^T(\boldsymbol{\Sigma}_{\boldsymbol{\beta}}^0)^{-1}(\boldsymbol{\mu}_{\boldsymbol{\beta}}^0).$$

Note that here the additional subscript $\boldsymbol{N}$ indicates the dependence on the 'new variational parameters' which are estimated in the M-step, while subscript $\boldsymbol{O}$ indicates the dependence on the 'old variational parameters'.

Estimation of the variational parameters - The M-step

Since $Q(\boldsymbol{N}|\boldsymbol{O})$ separates in two functions of which one only depends on $\boldsymbol{a}_{(\boldsymbol{N})}$ and the second only depends on $\boldsymbol{b}_{(\boldsymbol{N})}$, it can be shown that

$$\left(\boldsymbol{a}_{(\boldsymbol{N})}\right)^2 = \text{diagonal}\left(\boldsymbol{W}\left(\boldsymbol{\Sigma}_{\boldsymbol{\alpha},\boldsymbol{O}} + \boldsymbol{\mu}_{\boldsymbol{\alpha},\boldsymbol{O}}\boldsymbol{\mu}_{\boldsymbol{\alpha},\boldsymbol{O}}^T\right)\boldsymbol{W}^T\right) \quad (4)$$

$$\left(\boldsymbol{b}_{(\boldsymbol{N})}\right)^2 = \text{diagonal}\left(\boldsymbol{X}\left(\boldsymbol{\Sigma}_{\boldsymbol{\beta},\boldsymbol{O}} + \boldsymbol{\mu}_{\boldsymbol{\beta},\boldsymbol{O}}\boldsymbol{\mu}_{\boldsymbol{\beta},\boldsymbol{O}}^T\right)\boldsymbol{X}^T\right). \quad (5)$$

References

1. Jaakkola TS, Jordan MI. Bayesian logistic regression: a variational approach. Statistics and Computing. 2000;10(2):25–37.
