# Supplementary material for: A Variational Bayes Approach to the Analysis of Occupancy Models
Source: PLoS One. 2016 Feb 29;11(2):e0148966. doi: 10.1371/journal.pone.0148966 (PMC4771718; doi:10.1371/journal.pone.0148966)
Supplement: S3 Appendix — (PDF) [file pone.0148966.s004.pdf]

S4 Appendix

Convergence calculations.

The VB method is undertaken by maximising the lower bound to the marginal log-likelihood,

$$\mathbb{E}_q(\ln \underline{p}) - \mathbb{E}_q(\ln q(\boldsymbol{\alpha}, \boldsymbol{\beta})) - \mathbb{E}_q(\ln q(\boldsymbol{z})).$$

The VB algorithm is started by setting initial starting values for the parameters of the VB distributions as well as the variational parameters and are updated as the progresses from iteration to iteration. Denote the value of the lower bound at iteration t as A_t. Both algorithms are stopped when $|A_t - A_{t-1}| \leq \epsilon$ where $\epsilon = 10^{-6}$.
