# Supplementary figures and images for: A Variational Bayes Approach to the Analysis of Occupancy Models
Source: PLoS One. 2016 Feb 29;11(2):e0148966. doi: 10.1371/journal.pone.0148966 (PMC4771718; doi:10.1371/journal.pone.0148966)

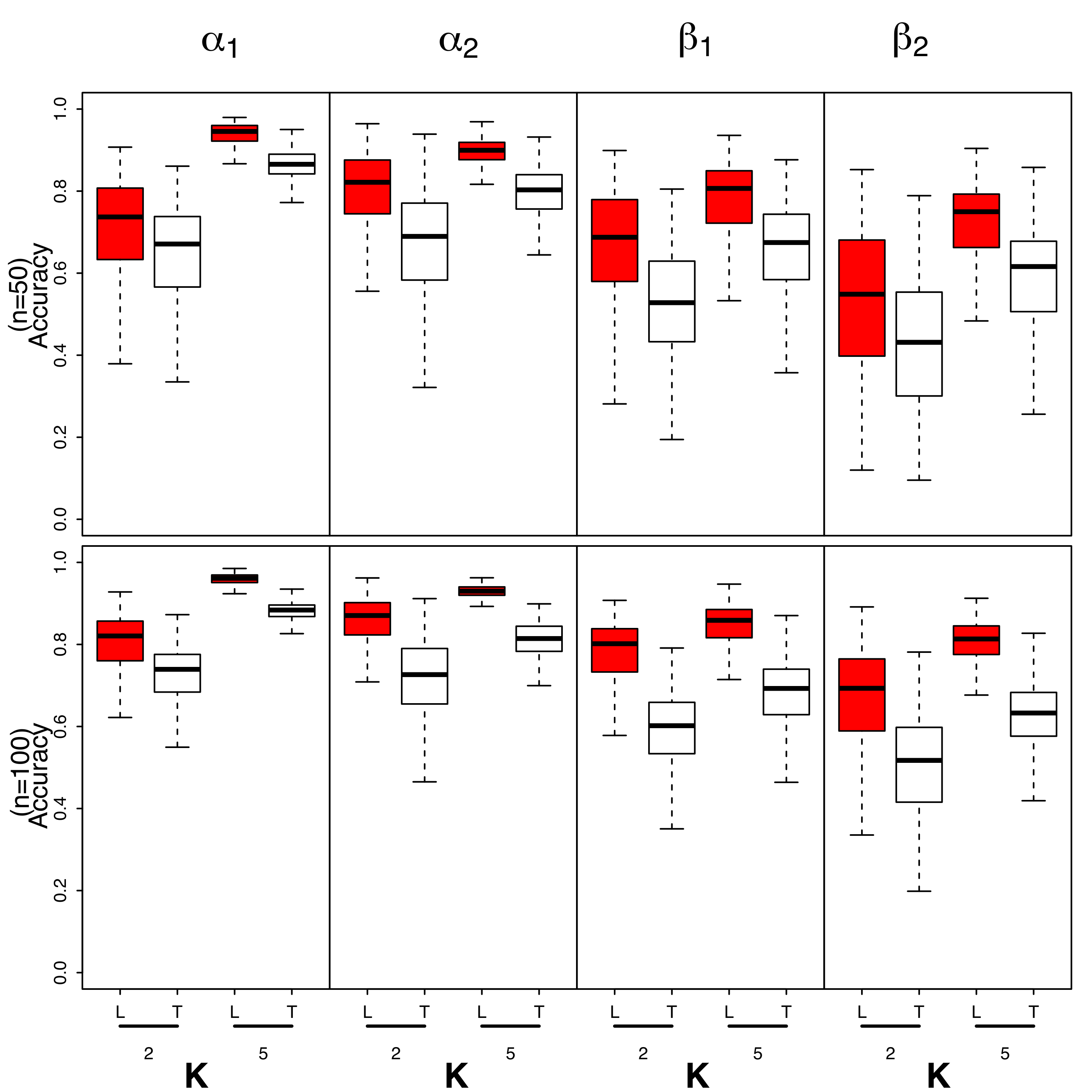

Supplement: S1 Fig — The detection probability is approximately 0.5 while the occupancy probability is approximately 0.3. (TIF) [file pone.0148966.s005.tif]

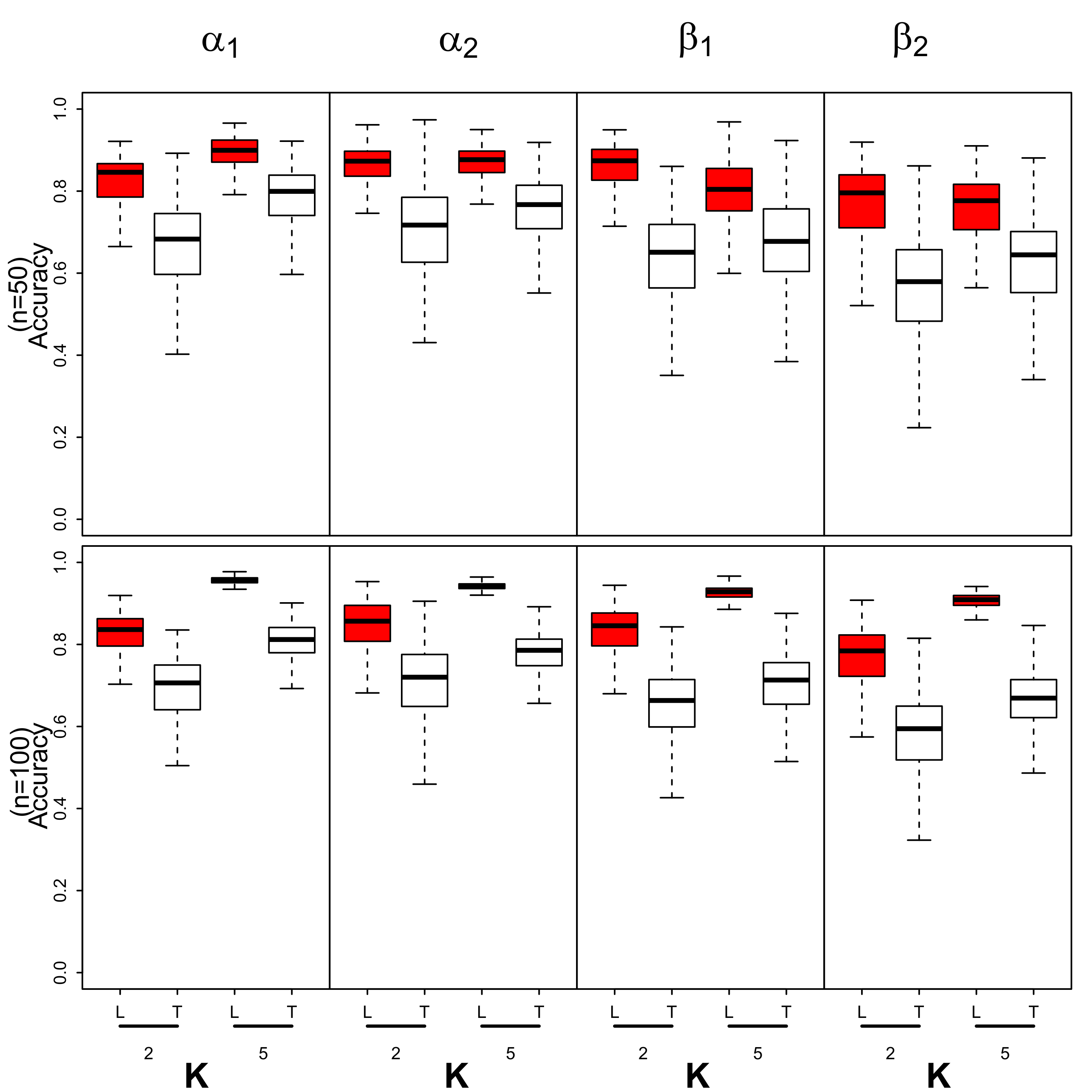

Supplement: S2 Fig — The detection probability is approximately 0.7 while the occupancy probability is approximately 0.3. (TIF) [file pone.0148966.s006.tif]

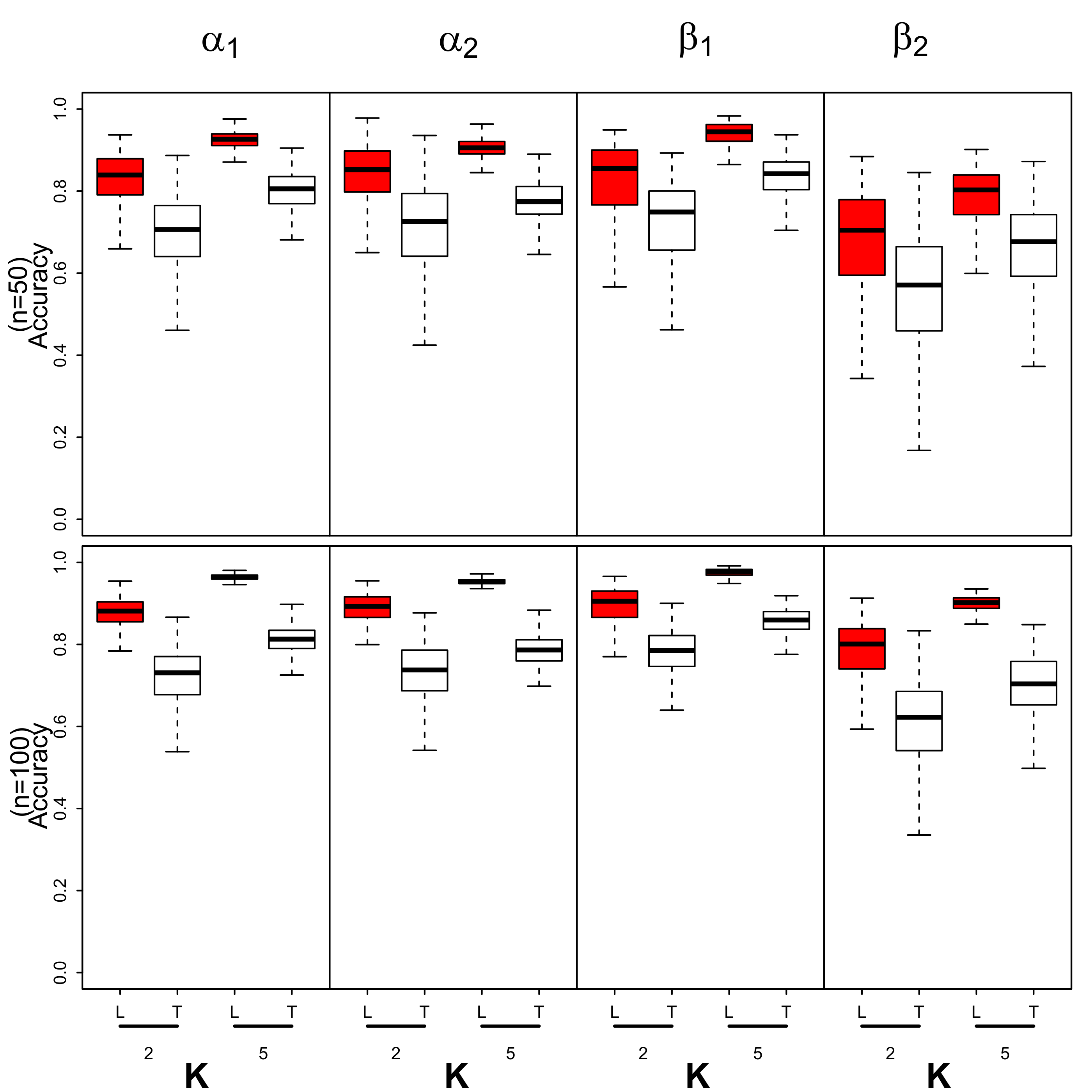

Supplement: S3 Fig — The detection probability is approximately 0.7 while the occupancy probability is approximately 0.5. (TIF) [file pone.0148966.s007.tif]
