# Supplementary material for: A Variational Bayes Approach to the Analysis of Occupancy Models
Source: PLoS One. 2016 Feb 29;11(2):e0148966. doi: 10.1371/journal.pone.0148966 (PMC4771718; doi:10.1371/journal.pone.0148966)
Supplement: S2 Code — (PDF) [file pone.0148966.s009.pdf]

1 VB Laplace approximation code

The VB Laplace approximation function and its usage is briefly explained below.

1.1 Function Call in R

The function call used to perform an analysis is as follows:

```
vb_model2_la(
formula, design_mats, alpha_0, beta_0,  Sigma_alpha_0, Sigma_beta_0,
LargeSample = FALSE, epsilon = 1e-05)
```

1.2 Named Arguments

The arguments of the function are as follows

- formula - Double right-hand side formula describing covariates of detection and occupancy in that order. e.g. Assume that the presence absence data is named y; the detection covariates is contained in a named list W (see below) and the occupancy covariates is stored X. Further suppose that the named lists are named W1, W2, W3 and X1 and X2 respectively. y $\sim$ W1 + W2 + W3 $\sim$ X1+ X2 would be one example of a suitable formula call. The function does not allow one to fit a model that only contains intercepts at the moment. This option will be included in future.

- design_mats - A named list generated by the call vb_Designs(W, X, y).

  W is a named list of data frames of covariates that vary within sites. i.e. The data frames are of dimension $n \times J$ where each row is associated with a site and each column represents a site visit.

e.g. Suppose W contained three data frames W1, W2 and W3; W$W1[1,] = the covariate values for site 1 for all of the visits. Note that some of the entries might be 'NA' meaning that no visit took place at those occasions.

X is a named data frame that varies at site level.

y is an $n \times J$ matrix of the detection, non-detection data, where n is the number of sites, J is the maximum number of sampling periods per site.

NOTE: THE FUNCTION DOES NOT ALLOW THERE TO BE ANY MISSING VALUES IN THE COVARIATE MATRICES IF A SURVEY WAS UNDERTAKEN AT A PARTICULAR LOCATION!!!

- alpha_0 - Prior mean of the detection covariate coefficients. It is assumed that the detection covariate coefficients have the following prior distribution $\boldsymbol{\alpha} \sim N(\text{alpha\_0}, \text{Sigma\_alpha\_0})$. Here $\boldsymbol{\alpha}$ is viewed as a vector.

- beta_0 - Prior mean of the occurrence covariate coefficients. It is assumed that the occupancy covariate coefficients have the following prior distribution $\boldsymbol{\beta} \sim N(\text{beta\_0}, \text{Sigma\_beta\_0})$. Here $\boldsymbol{\beta}$ is viewed as a vector.

- Sigma_alpha_0 - Prior covariance matrix of the detection covariate coefficients.

- Sigma_beta_0 - Prior covariance matrix of the occurrence covariate coefficients.

- LargeSample - LargeSample==TRUE - indicates that the number of sites is 'large' and that an approximation to $B(\mu, \sigma^2)$ is used instead of integrations (otherwise numerical integrations are performed).

- epsilon - Convergence measured relative to this quantity.

1.3 The values outputted by the function

- alpha - The VB estimate of the posterior mean vector of $\boldsymbol{\alpha}$. ($s \times 1$ vector)
- beta - The VB estimate of the posterior mean vector of $\boldsymbol{\beta}$. ($r \times 1$ vector)
- Sigma_alpha - The VB estimate of the posterior covariance matrix of the α vector. ($s \times s$ matrix)
- Sigma_beta - The VB estimate of the posterior covariance matrix of the $\boldsymbol{\beta}$ vector. ($r \times r$ matrix)
- occup_p - The VB estimate of the posterior occupancy probabilities at the sites considered. ($n \times 1$ vector)
- Log_mla - The lower bound of the log marginal log likelihood.
- Breakcounter - Breakcounter==1 if the number of iterations to perform the calculations are large. At the moment 'large' is viewed as 2000 iterations.

2 A small simulated data set

The following R code could be used to produce a small simulated data set that could be used to undertake the VB Laplace approximations.

```
#A simple example of how to construct y, X and W; the
#detection/nondetection data, site covariates and observation covariates
#-----------------------------------------------------------------------

require(MASS)
set.seed(1000)

beta.param = c(-1.85, 1.5, -0.5)
```

```
n = 5

#create 2 site covariates used to model occupancy
x1 = runif(n, -2,2)
x1 = (x1 - mean(x1)) / sd(x1)
x2 = runif(n, -5,5)
x2 = (x2 - mean(x2)) / sd(x2)
X = cbind(rep(1,n), x1, x2)
psi = as.vector(1/(1+exp(-X %*% beta.param))) ##logistic link function used
z = rbinom(n, size=1, prob=psi)

J = 3 #the maximum number of surveys (some sites might have fewer visits)

#three observation covariates used to model the detection probs
alpha.param = c(-1.35, 1.0, 0.5, -.25)
w1 = runif(n*J, -5,5)
w1 = (w1 - mean(w1)) / sd(w1)
w2 = runif(n*J, -1,1)
w2 = (w2 - mean(w2)) / sd(w2)
w3 = runif(n*J, 0,5)
w3 = (w3 - mean(w3)) / sd(w3)
W = array(dim=c(n,J,4))
W[,,1] = 1
W[,,2] = w1
W[,,3] = w2
W[,,4] = w3

p = matrix(nrow=n, ncol=J)
y = matrix(nrow=n, ncol=J)
for (j in 1:J)
{
p[, j] = c(1/(1+exp(-W[,j,] %*% alpha.param)))
y[, j] = rbinom(n, size=1, prob=z*p[, j])
}
#-----------------------------------------------------------------

#Now lets simulate the number of visits to each of the sites
#i.e. we need to set some of the y and W entries equal to
#NA
```

```
nvisits<-sample(1:J, n, replace=T)
empty.sites<-which(nvisits!= J)

for (i in 1:length(empty.sites))
{
#adds NA to sites with visits less than J
y[ empty.sites[i], (nvisits[empty.sites[i]]+1):J ] <- NA

#adds NA to W entries with visits less than J
W[ empty.sites[i], (nvisits[empty.sites[i]]+1):J, ] <- NA
}

#Note W[i,,] are the covariate values for site i
#each row is for a specific visit
#-----------------------------------------------------------------

#An nxJ matrix of the observed measured data,
#where n is the number of sites and J is the
#maximum number of observations per site.
Y.eg<-y
#-----------------------------------------------------------------

#siteCovs
#A data.frame of covariates that vary at the site level.
#This should have n rows and one column per covariate
X.eg=as.data.frame(cbind(x1,x2))
#-----------------------------------------------------------------

#obsCovs
#the obsCovs matrix is constructed as per the 'unmarked' package
#i.e. W.eg.l1 is a named list of data.frames of covariates that
#vary within sites.
#i.e. The dataframes are of dimension n by J
#where each row is associated with a site
#and each column represents a site visit.
#e.g. W.eg.l1$W1[1, ] = the covariate values for site 1 for all of the
#visits. Note that some of the entries might be 'NA'
#meaning that no visit took place at those occasions.
```

```
W1=matrix(NA,nrow=n, ncol=J)
W2=matrix(NA, nrow=n, ncol=J)
W3=matrix(NA, nrow=n, ncol=J)
for (i in 1:n)
{
W1[i,]<- W[i,,2]
W2[i,]<- W[i,,3]
W3[i,]<- W[i,,4]
}

#colnames(W1)<-paste("W1.",1:J,sep="")
#colnames(W2)<-paste("W2.",1:J,sep="")
#colnames(W3)<-paste("W3.",1:J,sep="")

W.eg.l1<-list(W1=W1, W2=W2, W3=W3)
W.eg.l1
#------------------------------------------------------------------

#An alternate way of 'viewing' the site covariates is as follows:
#Create a list element; one for each site, where the data
#for each site have been stacked one below the other either as
#a dataframe or as a matrix. e.g.
#W.eg.ls[[2]] is the data for site 2.

W.eg.l2=list(list())

for (i in 1:n)
{
if (nvisits[i]!=1)
{
dframe<-as.data.frame(W[i,1:nvisits[i],][,-1])
}else
{
dframe<-as.data.frame(matrix(W[i,1:nvisits[i],][-1],nrow=1))
}

names(dframe)<-c("w1","w2","w3")
W.eg.l2[[i]]<-dframe
}
```

```
#------------------------------------------------------------------

#Two different ways of representing the observation covariates
W.eg.l1
W.eg.l2
#------------------------------------------------------------------

#If the site covariates are provided as per W.eg.l1
#then we can construct W.eg as follows
#(here W.eg is the way in which 'vb_model2_la')
#creates the site covariate matrix W)
#We assume that all sites are visited at lest once
#although all might not be visited J times
#We further assume that there are no missing covariate
#values for those occasions sites are visited

W.temp<-NULL
n<-length(W.eg.l1)
for (i in 1:n)
{
W.temp<-cbind(W.temp, W.eg.l1[[i]])
}
W.temp

nvisits<-apply(W.eg.l1[[1]],1,function(x){length(na.omit(x))})
nvisits

W.eg<-NULL
for (i in 1:n)
{
W.eg<-rbind(W.eg, matrix( c(na.omit(W.temp[i,])), nrow=nvisits[i]) )
}
W.eg

#------------------------------------------------------------------

#If the site covariates are provided as per W.eg.l2
#then we can construct W.eg as follows
W.eg<-NULL
```

```
n <-length(W.eg.l2)
for (i in 1:n)
{
W.eg<- rbind(W.eg, W.eg.l2[[i]])
}
W.eg
#-------------------------------------------------------------------

SimData<-list(y=Y.eg, X=X.eg, W.eg.l1=W.eg.l1, W.eg.l2=W.eg.l2, W_vb=W.eg)
```

The simulated data is stored in a list named SimData and it's contents are displayed below.

```
> SimData
$y
     [,1] [,2] [,3]
[1,]    0   NA   NA
[2,]    0    1    0
[3,]    0   NA   NA
[4,]    0    1    0
[5,]    0    0   NA

$X
          x1         x2
1 -0.5802233 -1.0949354
2  1.0468539  1.3166989
3 -1.3879419  0.7589495
4  0.7897816 -0.5628716
5  0.1315297 -0.4178415

$W.eg.l1
$W.eg.l1$W1
           [,1]       [,2]       [,3]
[1,] -1.1475341         NA         NA
[2,]  0.3516124  1.4671572  0.2061681
[3,]  0.8607333         NA         NA
[4,] -1.1047705 -1.1515037 -1.0836617
[5,]  0.6777174  0.5505103         NA
```

```
$W.eg.l1$W2
            [,1]       [,2]       [,3]
[1,]  0.08380091         NA         NA
[2,] -1.61591051 -1.7122869 -0.0359153
[3,]  0.06444337         NA         NA
[4,]  1.39241296  0.1392068  0.2994426
[5,] -0.10518068 -0.7375655         NA

$W.eg.l1$W3
           [,1]       [,2]      [,3]
[1,]  0.6508060         NA        NA
[2,]  0.6021102  0.5024673 0.6489031
[3,] -0.0692492         NA        NA
[4,] -1.5268438 -0.2837397 1.4945716
[5,] -0.3372867  0.2584308        NA


$W.eg.l2
$W.eg.l2[[1]]
         w1         w2       w3
1 -1.147534 0.08380091 0.650806

$W.eg.l2[[2]]
         w1         w2        w3
1 0.3516124 -1.6159105 0.6021102
2 1.4671572 -1.7122869 0.5024673
3 0.2061681 -0.0359153 0.6489031

$W.eg.l2[[3]]
         w1         w2         w3
1 0.8607333 0.06444337 -0.0692492

$W.eg.l2[[4]]
         w1        w2         w3
1 -1.104771 1.3924130 -1.5268438
2 -1.151504 0.1392068 -0.2837397
3 -1.083662 0.2994426  1.4945716

$W.eg.l2[[5]]
```

```
          w1         w2         w3
1 0.6777174 -0.1051807 -0.3372867
2 0.5505103 -0.7375655  0.2584308


$W_vb
           w1          w2         w3
1  -1.1475341  0.08380091  0.6508060
2   0.3516124 -1.61591051  0.6021102
3   1.4671572 -1.71228685  0.5024673
4   0.2061681 -0.03591530  0.6489031
5   0.8607333  0.06444337 -0.0692492
6  -1.1047705  1.39241296 -1.5268438
7  -1.1515037  0.13920676 -0.2837397
8  -1.0836617  0.29944258  1.4945716
9   0.6777174 -0.10518068 -0.3372867
10  0.5505103 -0.73756554  0.2584308
```

3 A small example

The following R code could be used as an example of how to use the VB code in order to undertake a small analysis.

```
## Load the  data into your workspace
##-------------------------------------

#This data set is stored as a supplementary information document
#First download the file and then save it into your working directory
#before running the rest of the script
load("S2_Data.rda")

#Set Uninformative priors
#-----------------------
#Coefficients in the detection model
alpha_0 <- matrix(0, ncol=1, nrow=4)
#Covariance matrix of the coefficients in the detection model
```

```
Sigma_alpha_0 <- diag(4)*1000
#Coefficients in the occupancy process
beta_0 <- matrix(0, ncol=1, nrow=3)
#Covariance matrix of the coefficients in the occupancy model
Sigma_beta_0 <- diag(3)*1000

#Construct the required matrices using vb_Designs
#------------------------------------------------
#Ensure that the function 'vb_Designs' is stored in the workspace
#The function is included here if this was not done

vb_Designs<-function(W, X, y)
{
   #create the required 'response' and 'regressor matrices'
   #using all of the X and W data
   #the output is stored as a named list

   #create the Y matrix that will be used
   Y<-matrix(na.omit(matrix(t(y), ncol=1)))
   pres_abs <- apply(y,1,max,na.rm=T) #check if this will work for NA's

   #create the W matrix
   W.temp<-NULL
   nv<-length(W)

   for (i in 1:nv){W.temp<-cbind(W.temp, W[[i]])}

   nvisits<-apply(W[[1]],1,function(x){length(na.omit(x))})
   n<-length(nvisits)

   W.out<-NULL
   for (i in 1:n)
   {
      W.out<-rbind(W.out, matrix( c(na.omit(W.temp[i,])), nrow=nvisits[i]) )
   }
   colnames(W.out)<-names(W)

   list(Y=as.data.frame(Y), X=as.data.frame(X), W=as.data.frame(W.out),
        Names=c( colnames(X), colnames(W.out)), nvisits=nvisits,
```

```
        pres_abs=pres_abs)
}
design_mats<-vb_Designs(W=SimData2$W.eg.l1, X=SimData2$X, y=SimData2$y)

#Here we use the large sample approximation and run the VB algorithm
#--------------------------------------------------------------------
#Assume that the formula used will be of the following form:
#formula1<- y~X1+X2~W1+W2+W3
#The occupancy model uses 2 covariates, X1 and X2; while
#the detection model uses 3 covariates W1, W2 and W3
#Intercepts are included in both models
#The function does not allow one to repress the intercept term

#ensure that the 'vb_model2_la' function is in the workspace
vb_fit<-vb_model2_la(y~X1+X2~W1+W2+W3, design_mats=design_mats,
alpha_0=alpha_0, beta_0=beta_0,
Sigma_alpha_0=Sigma_alpha_0, Sigma_beta_0=Sigma_beta_0,
LargeSample=TRUE, epsilon=1e-5)

#The detection model parameters
vb_fit$alpha

#The occupancy model parameters
vb_fit$beta

#The respective covariance matrices
vb_fit$Sigma_alpha
vb_fit$Sigma_beta

#The approximate conditional ocupancy probabilities
plot(vb_fit$occup_p, ylab="Occupancy prob", xlab="Site number")
```
